# Genetic variation and synonymous cultivars in the USDA lychee (*Litchi chinensis* Sonn.) collection assessed using genome-wide SNPs

**DOI:** 10.1007/s10722-025-02406-y

**Published:** 2025-03-18

**Authors:** Joseph Rootkin, Grace Harrison-Tate, Carol R. Mayo-Riley, Tracie Matsumoto, Mark A. Chapman

**Affiliations:** 1 Biological Sciences, University of Southampton, Life Sciences Building 85, Highfield Campus, Southampton, SO17 1BJ UK; 2 Tropical Plant Genetic Resources & Disease Research Unit, Daniel K. Inouye U.S. Pacific Basin Agricultural Research Center, 64 Nowelo Street, 928 Stainback Highway, Hilo, HI 96720 USA

**Keywords:** Lychee, Genetic variation, Germplasm collections, Synonymous cultivars, Admixture

## Abstract

Lychee (*Litchi chinensis*) fruits have high nutritional and pharmacological value, yet the crop is underutilized on global markets partly due to cultivation problems outside the native area and lack of knowledge/misidentification of cultivars. Using reduced representation sequencing, 78 trees representing 57 cultivars plus 13 trees without varietal names from the USDA lychee collection on Hawai’i were analysed. We aimed to (1) understand the distribution of genetic variation, (2) ensure individuals of the same named variety were indeed likely the same cultivar, and (3) to identify cases of cultivar synonymy. After removing single nucleotide polymorphisms (SNPs) with substantial missing data, rare alleles and low confidence SNPs, analysis of ca. 10,400 SNPs was carried out. Genetic clustering and phylogenetic analysis identified two main groups which, based on existing data, represent the late and extremely early maturing cultivars, with many admixed individuals likely representing those with intermediate maturity times. A core set of 12 SNPs for cultivar identification were identified. Trees from the same named variety were not always closely related, representing misidentification. Similarly, some trees with different varietal names appear to be the same (i.e., synonymous cultivars); in some cases, this backed up existing knowledge about the transport and renaming of cultivars between countries. Based on genetic similarity, we found 13 groups of clones varying in size from 2–9 individuals. Fifty-five of the 91 trees (60%) were in these groups. Together, our data has the potential to inform the conservation of lychee accessions and backs up previous work that there are primarily two domesticated gene pools with a third group of accessions being admixed. Going forward, our results could lead to the improvement of lychee, a step in making it a staple crop.

**Supplementary Information:**

The online version contains supplementary material available at 10.1007/s10722-025-02406-y.

## Introduction

Currently, 690 million people worldwide do not have enough food to meet their daily energy requirements, and 2 billion suffer from micronutrient deficiencies (WHO 2021). With the global population expected to surpass 9 billion by 2050 (Godfray et al. 2010), food security is of paramount concern. Our reliance on few staple crops is expected to be insufficient to meet the food demand of the future population; 90% of our calories come from just 15 crops (FAO 2017) whereas 7,000 plant species have been used as crops in human history (FAO 2010). It is therefore vital that we tap into the potential of underutilised crops to help solve future food scarcity and malnutrition.

Underutilised crops, also known and minor, indigenous, and orphan crops, are usually those which are not achieving their full potential, due to lower yield, the presence of antinutrients, or other issues compared to widespread staple crops (Mayes et al. 2012). Their potential in terms of climate resilience, nutrients and other factors is often high but under-researched (Chapman 2022). Improving our knowledge of genetic relationships in underutilised crops is an important step in realising the potential of these crops (Chapman et al. 2022). The identification of adaptive traits (e.g., drought resistance, high nutrient concentration, yield) as well as those that are limiting their use (e.g., antinutrient content, susceptibility to pathogens) and understanding their genetic basis, provides insights into the crop origin and evolution (Ross-Ibarra et al. 2007). These analyses can facilitate efficient selection/breeding, allowing for the development of cultivars with desirable characteristics.

Due to limited financial resources, which of the hundreds of underutilised crops to prioritise for breeding and improvement is an important question (Azam-Ali 2010). Understanding the genetic variation in underutilised crop species is an important step in this prioritisation. High genetic variation within a species may reveal under-sampled adaptive traits that can be harnessed for genetic improvement of the crop, and a close relationship to other crops is highly beneficial, setting the stage for shared information and resources to speed up the identification of genes underlying breeding traits. Further, the agricultural potential, consumer demand and nutritional value are equally as important. One crop that fits these criteria is lychee (*Litchi chinensis* Sonn.), a tropical fruit species with high genetic variation (Liu et al. 2015, 2023). Based on SNP (single nucleotide polymorphism) markers, genetic structure in litchi matches separation into different maturity groups (from extra early to late), with cultivars that have an intermediate maturity phenotype tending to be admixed between early and late maturing groups (Liu et al. 2015, 2023). Lychee has a close genetic relationship to other tree crops (longan [*Dimocarpus longan* Lour.] and rambutan [*Nephelium lappaceum* L.]), and has high nutrient content (vitamin C and antioxidants) (Wall 2006), yet currently does not meet its potential, especially compared to other fruit crops grown in the same region such as apples, pears and citrus (FAO, 2020).

**Fig. 1:**
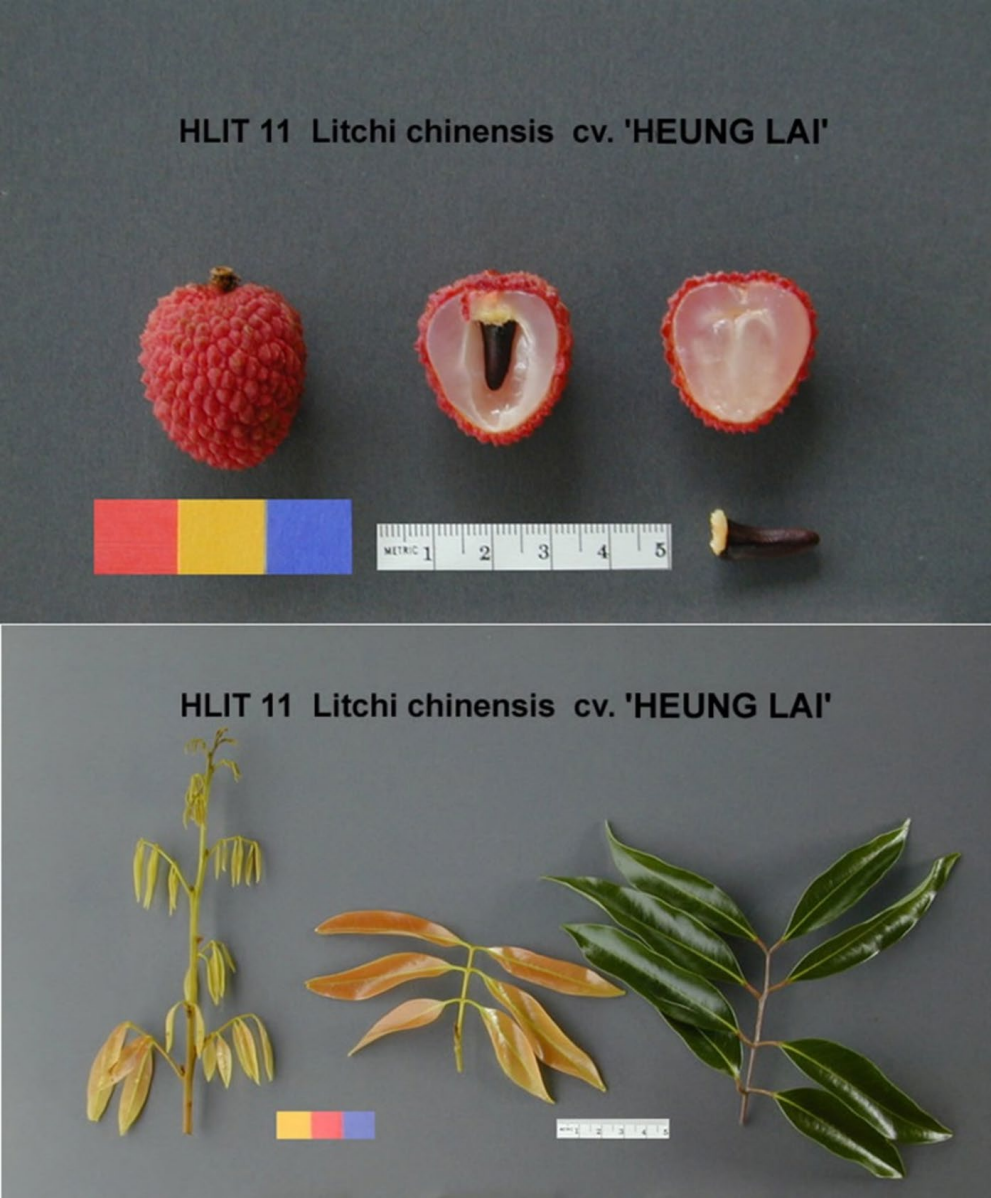
Fruit (top) and vegetative (bottom) morphology of the lychee cultivar ‘Heung Lai’

Lychee (Fig. 1) is a prominent member of the Sapindaceae and closely related to longan (*Dimocarpus longan* Lour.). Lychee are medium to large, evergreen trees with dense foliage and round canopy that can reach 9 to 30 m in favourable conditions. Leaves are arranged alternatively and are pinnately compound with 2 to 5 pairs of leaflets which are soft and leathery to the touch. The inflorescence is a branched panicle with hundreds of small white or yellowish flowers (Chia et al. 1997). Fruits are usually single seeded, pendent clusters of two to 30 fruits that are round, ovoid or heart shaped depending on the cultivar; the edible flesh surrounding the seed is the aril (Chapman 1984; Morton 1987). Lychees are relatively drought tolerant when vegetative but drought after flowering will result in reduced fruit production (Menzel and Simpson 1990).

Lychee trees are native to the region between southern China, northern Vietnam, and Myanmar (Menzel 2002), but primarily and historically cultivated in Southern China, with relatively recent cultivation across the whole of Asia. Lychee has been cultivated in China since the second century BC, and the first publication devoted to lychee cultivars was in 1059 AD (Hu et al. 2022). The tree arrived in Burma by the end of the seventeenth century and was introduced to India 100 years later. It reached Madagascar, Mauritius, and Florida in the 1870s, California by 1897 and Australia by 1954. The propagation attempts in Australia, South Africa, and the U.S.A have been unsuccessful in comparison to cultivation in Asia (Menzel and Simpson 1987), meaning there is scope for improvement.

Lychee domestication took place twice in geographically distinct parts of China from divergent wild populations, resulting in two main gene pools that differ in maturity time (Hu et al. 2022). Yunnan-originating accessions are extremely early maturing cultivars (EEMCs) and Hainan-originating ones are late-maturing (LMCs). Cultivars with better fruit quality group in the LMCs instead of the EEMCs. Hybrids, likely produced deliberately during cultivation, are intermediate and known as early maturing cultivars (EMCs) (Liu et al. 2015; Hu et al. 2022). Hu et al. (2022) identified a *CONSTANS-like* gene, *COL307*, through association analysis, as likely playing a role in the difference in seasonal flowering time, and therefore fruit maturation time, between the EEMCs and LMCs. A deletion in the EEMCs reducing gene expression relative to the copy in the LMCs. EMCs are heterozygous for this deletion, and the genotype at this locus can be detected using a simple PCR (polymerase chain reaction) procedure (Hu et al. 2022).

The fruit is highly regarded for its sweet taste and its involvement in traditional Chinese trade, history, and medicine (Ibrahim and Mohamed 2015). In the Chinese Tang dynasty (1,300–1,100 years before present [YBP]) the emperor set up a courier service for lychee transportation from the south to the courts in the north (Hu et al. 2022). Lychee trees have the longest productive lifespan of tropical and subtropical fruit trees, the oldest lychee tree ‘Songxiang’ is 1,250 years old and still bearing fruit (Hu et al. 2022).

There have been several investigations analysing the nutritional and health benefits of lychee. The fruit is high in antioxidants, potassium, copper, vitamin B2 and vitamin C; lychees have been reported to contain more vitamin C than oranges, strawberries, and pineapples (34.7 mg/100g; Cabral et al. 2014). The flesh and peel have high antioxidant levels which inhibit the generation of free radicals, combating the effects of reactive oxygen species induced oxidative damage, antioxidants isolated from lychee flowers inhibited Cu2 + induced human LDL oxidation (Yang et al. 2012). Reported health benefits include promoting hair growth, preventing skin aging, promoting cardiovascular health, prevention of cataracts, anti-inflammatory effects, strengthening bones, improving digestion, and preventing anaemia (Zhao et al. 2020; Sun et al. 2021).

Despite global lychee production reaching 3.5 M tonnes in 2018 (Mitra and Pan 2020), the lychee market is dwarfed by other tropical fruits, for example banana (125 M tonnes in 2021) and mangoes, guavas and mangosteens (57 M tonnes combined) (FAO 2020). The gap in markets may seem unclear given that lychee have been cultivated and traded for over 4,000 years (Hu et al. 2022), however, lychee production is riddled with unpredictability, flowering is conditional upon cool day condition for induction including because of climate change (Nath et al. 2018). Over 80% of fruit is produced within a short period from early June to mid-July, the fruit spoils easily so a fresh, year-round supply is almost impossible (Hu et al. 2022). Cultivars grown outside of China often suffer from irregular fruiting periods and poor fruit quality; a well-managed orchard in China can produce 15 tonnes/ha, while average production in Thailand and Vietnam is 3.5 and 2.0 tonnes/ha/year, respectively (Menzel 2002). The large gap between average and potential yields stems from inadequate farming techniques, lychee erinose mites (which damage the inflorescence and reduce output), poor cultivar selection, poor soil quality, irregular fruiting periods and over-crowding of trees (Menzel and Simpson 1987). Better knowledge of cultivars and their agronomic traits can improve yield, and a robust assessment of genetic and cultivar variation is needed for this.

Over 400 lychee cultivars are preserved at the National Lychee Germplasm Resources in Guangzhou, China (Liu et al. 2015) with a range of properties regarding taste, fruit size, fruit maturation time and optimal growing conditions. The US Department of Agriculture (USDA) has 94 accessions growing in Hawai’i (USDA 2018). A key consideration when investigating the genetic diversity of lychee, and any crop, is the issue of synonymous and homonymous cultivars (Liu et al. 2015). The former are identical accessions (clones) with different names, and the latter are genetically different accessions under the same cultivar name. Identifying these inconsistencies is important for efficiently preserving genetic diversity. Identification of a core set of SNP markers would help expedite the identification of clones going forward, as has been done in other tropical trees (for example coffee; Zhang et al. 2021).

Identifying these is not a trivial task – using a small number of markers means the chance of identifying apparently identical samples is high, even when diversity is present, for example in research of grape (Riaz et al. 2013) and apple (Baric et al. 2009) where only a few SSR markers were employed. In jujube (*Ziziphus jujuba* Mill.), 114 cultivars were analysed with 192 SNP markers and 17 groups of synonymous cultivars were identified among these (Song et al. 2021). In research using a very large numbers of markers, where high throughput reduced representation sequencing is used, it is typical that samples known to be identical do not resolve as genetically identical (e.g. Carvajal-Yepes et al. 2023; Villano et al. 2023). This can be due to a range of technical factors, for example the miscalling of heterozygotes, sequencing errors and inconsistencies in the restriction enzyme digestion (Hamblin and Rabbi 2014). In cassava (*Manihot esculenta* Crantz), two investigations carrying out the laboratory protocol in duplicate for a subset of samples resulted in non-identical genotypes, which were used to standardise the data to identify synonymous cultivars (Rabbi et al. 2015; Carvajal-Yepes et al. 2023). In addition, minor variations among plants within a named cultivar can be present, especially if somatic mutations arise and then are maintained clonally; these are sometimes called ‘sports’ (Cabezas et al. 2011). This could be common in lychee as cultivars are maintained via clones.

Due to trade across language and geographic boundaries, cultivar names can get misconstrued until two separate names for the same cultivar exist (Menzel 2002). Similarly, there is a common practice of giving one cultivar two different names in different countries, for example, ‘Tai So’ lychee is the Thai name for ‘Mauritius’ from the Philippines, a clear example of synonymy. In a previous analysis, two pairs of accessions were identified as synonyms using 90 SNP markers (Liu et al. 2015). As a small number of markers were used, these should be followed up. Further, because cultivars are traditionally identified by eye using morphological keys, two morphologically similar cultivars might not be genetically identical (homonyms) because morphology can be influenced by environmental conditions (Yee 1976). In lychee there is no clear relationship between morphological traits (such as skin segmentation or fruit size) and genetic relationships (Liu et al. 2015).

The aim of this work is to characterise genetic diversity in the USDA lychee collection using reduced representation sequencing, to compare our findings to analyses of South-East Asian accessions discussed above (e.g., Liu et al. 2015; Hu et al. 2022) and to identify synonymous and homonymous accessions. The collection consists of 94 accessions from 69 named cultivars and differs from those used in Hu et al. (2022) and Liu et al. (2015) in that it only includes cultivated accessions, most of which have not previously been genetically characterized. Thirteen accessions in the collection had not been assigned a cultivar name. Accessions have been sourced from China, Thailand, Australia, Taiwan, and the USA.

Improving our understanding of genetic population structure by identifying and alerting to misidentifications can (1) increase genetic variation in germplasm collections, improving the potential to respond to future challenges such as emerging diseases, (2) make for more efficient germplasm storage by not storing duplicate samples, (3) clarify parental combinations and lineages, aiding the performance of breeding programmes, and (4) avoid stratification and admixture in genome wide association research, improving the accuracy and reliability of results (Liu et al. 2015). Going forward, for improving the potential of lychees it is imperative that lychee cultivars are classified by genetic markers over morphology to create an undisputable identification system.

## Materials and methods

### Samples and DNA extraction

Ninety-one of the 94 USDA accessions were analysed in this study (Online Resource 1). 78 of these represent one of 57 named cultivars (1–7 samples per cultivar) and 13 do not have cultivar names (hereafter ‘unknown’) (see USDA 2018). Accessions in this collection represent semidomesticated and domesticated cultivars grown throughout the world. A large focus of the collection is plant material that are productive in the growing conditions of Hawaii, Florida, and Puerto Rico (USDA 2018).

DNA was extracted from dried leaves of the 91 trees using a modified version of the (Doyle and Doyle 1990) CTAB-based extraction protocol. Modifications include incubating samples for 45 min to 1 h instead of 30 min, extracting with chloroform:isoamyl alcohol (24:1) twice instead of once, increasing precipitation time with cold isopropanol to 2 h, and performing two rounds of washing with ethanol (70% and 100%). DNA samples were then quantified on a nanodrop spectrophotometer and quality checked with agarose gel electrophoresis.

### Reduced representation sequencing

We used Specific-Locus Amplified Fragment Sequencing (SLAF-Seq) (Sun et al. 2013) to provide genome-wide SNPs to analyse genetic diversity and relationships. SLAF-seq was performed by BMKgene (Münster, Germany). Reads were mapped to the lychee cv. Feizixiao genome (Hu et al. 2022), using BWA-MEM ver. 0.7.10-r789 (Li and Durbin 2009). Local realignment around indels was performed using RealignerTargetCreator and IndelRealigner in GATK (McKenna et al. 2010). Since different variant calling pipelines may be prone to unique biases and provide inconsistent results, we called variants using both the mpileup command in SAMtools v1.9 (Li et al. 2009) and GATK UnifiedGenotyper with default settings. Variants identified by both were selected using the SelectVariants package in GATK and filtered using the GATK "Best Practices" workflow. Data was exported in VCF format.

### Bioinformatics

We used VCFtools (Danecek et al. 2011) to carry out a range of filtering steps to assess the effect of two parameters. We filtered for SNPs with less than 5, 10 or 20 samples with missing genotype calls and removed SNPs potentially within linkage disequilibrium (distance of 2kb or 10kb); six combinations were therefore examined. For all, we excluded SNPs with minor allele frequency < 5%.

For all six sets of filtered SNPs, we assessed the phylogenetic relationships between accessions to determine the effect of missing data and physically linked SNPs (potentially in LD) on the identification of relationships among accessions. From the filtered VCF files, VCF2Dis (https://github.com/BGI-shenzhen/VCF2Dis) was used to output distance matrices which were uploaded to FastMe 2.0 (Lefort et al. 2015) and analysed with the NJ algorithm (Saitou and Nei 1987). Trees were converted to newick format and opened in ITOL (Letunic and Bork 2021) for improving the appearance.

After selecting one of the filtered datasets (see results) we carried out the following analyses:

#### Phylogenetic analysis

For phylogenetic analysis we used VCF2Dis to generate 1000 bootstrapped matrices which were combined and turned into 1000 tree files using PHYLIP (Felsenstein 2004) command fneighbor (using http://emboss.toulouse.inra.fr/cgi-bin/emboss/fneighbor) and then bootstraps generated using the PHYLIP command fconsense (using http://emboss.toulouse.inra.fr/cgi-bin/emboss/fconsense). The tree was plotted in ITOL (Letunic and Bork 2021).

#### Bayesian clustering analysis

STRUCTURE (Pritchard et al. 2000) analysis was performed using the same chosen set of filtered SNPs. Plink version 1.07 (Purcell et al. 2007) was used to create the input file. STRUCTURE was run using a range of possible populations from 1 to 10, with five iterations of K for 50,000 MCMC repeats after a 20,000-repeat burn-in. The deltaK approach (Evanno et al. 2005) was used to find the optimal number of clusters. This was then plotted using the online server for CLUMPAK (Kopelman et al. 2015); http://clumpak.tau.ac.il/).

#### The link between maturation time and cultivar relationships

As mentioned above, the two main genetic groups within lychee are differentiated for maturity time, either being EEMC or LMC, with hybrid/admixed individuals being EMC. Previous cases of a cultivars known fruit maturation time (LMC/EMC/EEMC) were found in Hu et al. (2022) and Liu et al. (2015), and we used information in FAO (2002) to convert between the USDA cultivar names we used in this study and the Chinese names used in these other publications. We then used the primers presented in Hu et al. (2022) to examine additional samples for their genotype at the flowering time gene known to show differentiation between the EEMCs and LMCs. To do this, we used DNA samples extracted as above, diluted to 10ng/µl and standard PCR protocols (e.g., Chapman 2019). Based on Hu et al. (2022), EEMCs and LMCs show amplification with only one of the pairs of primers, with EMCs showing amplification with both pairs of primers.

#### Synonymous and homonymous accessions

Synonymous cultivars are named differently but are genetically the same, whereas homonymous cultivars are those with the same name but are in fact genetically different. There is not a universal measure of genetic distance which defines two samples belonging to the same cultivar, however 100% identical is not an appropriate cut-off when using highly multiplexed sequencing and large data (thousands of SNPs) such as ours (see introduction).

We aimed to identify a cut-off value which would allow us to identify synonymous and homonymous cultivars as well as identify potential cultivar names for the samples currently without varietal names. We first identified pairs of samples of the same cultivar name (or known synonymous cultivar pairs) that were sisters in the phylogeny, however only three such pairs were identified. We therefore expanded to include clusters of accessions where at least two identically named cultivars were found, with one or two other samples (named or unknown) within the group. The maximum distance between samples within these groups was then used as the cut-off to identify synonymous and homonymous cultivars.

#### A core SNP set

We used CoreSNP (https://github.com/admy55/CoreSNP) (Dou et al. 2023) to analyse the 91 samples and to identify a set of SNPs that could differentiate all individuals.

## Results

### Sequencing, phylogenetic analysis and genetic clustering

The raw genotyping data comprised an average of 2.12 M SNPs per individual and a total of 4,467,310 SNPs across samples with a quality score > 30 and a depth > 10. The majority of SNPs were C/T (22.80%) or G/A (22.82%) (Online Resource 2) and were present on all chromosomes but at variable density (Online Resource 3).

A large proportion of these SNPs were expected to have substantial missing data and/or be in LD, however as there is no general rule for how SNP thinning should be carried out, we examined six combinations of parameters, resulting in between 7250 and 20,792 SNPs being retained (Table 1).

**Table 1:** the number of SNPs retained after carrying out filtering with six combinations of two parameters

	Thin 2,000	Thin 10,000
Max missing 5%	8,992	7,250
Max missing 10%	14,072	10,379
Max missing 20%	20,792	13,961

We examined whether these different settings affected the topology of the NJ phylogenetic tree. All six trees (Online Resource 4) have very similar relationships with the only differences at internal nodes being (1) differences in the relative placements of the clades containing SCH1/2/3, KMV/UNK9/ITO and BOS/KMP1, and (2) differences in the placement of NMT2. Other minor differences were identified near the tips, but this would have no bearing on the identification of synonymous cultivars (see below). We therefore chose the dataset that resulted in the NJ tree that was the least different from others (thin 10k, missing 10%; 10,379 SNPs).

**Fig. 2:**
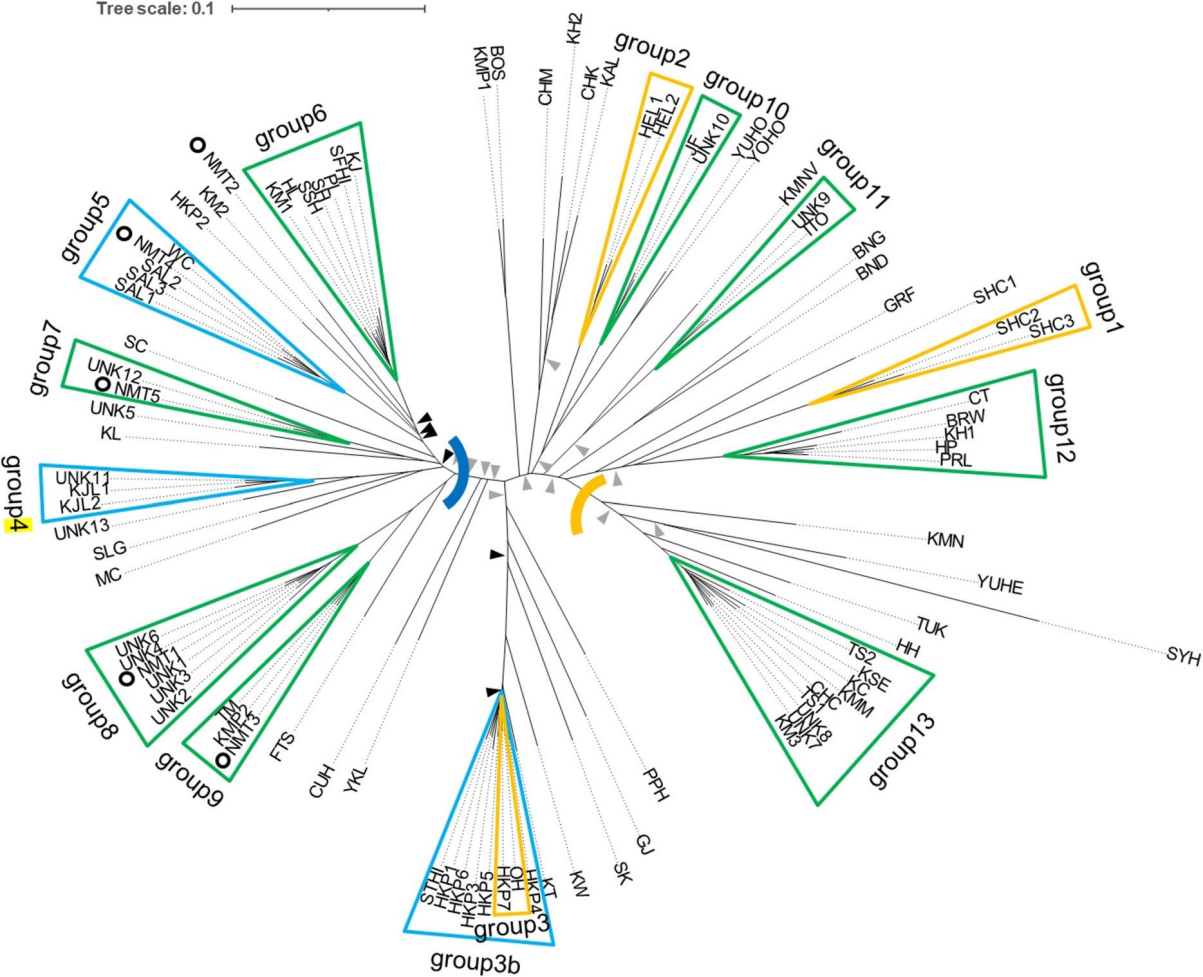
NJ phylogenetic tree of 91 lychee accessions. Accession codes are explained in Online Resource 1. Node support is 100% unless indicated with a black (70–99%) or grey arrow (< 70%). Node support on very short branches within very tight clusters is omitted. The blue and yellow curved lines indicate the individuals with > 80% to cluster 1 (blue curve) or cluster 2 (yellow curve) in the STRUCTURE analysis (Fig. 3). Groups identified in the text are indicated with coloured triangles and names. Accessions of cultivar No Mai Tsze are highlighted with circles (see text for details)

**Fig. 3:**
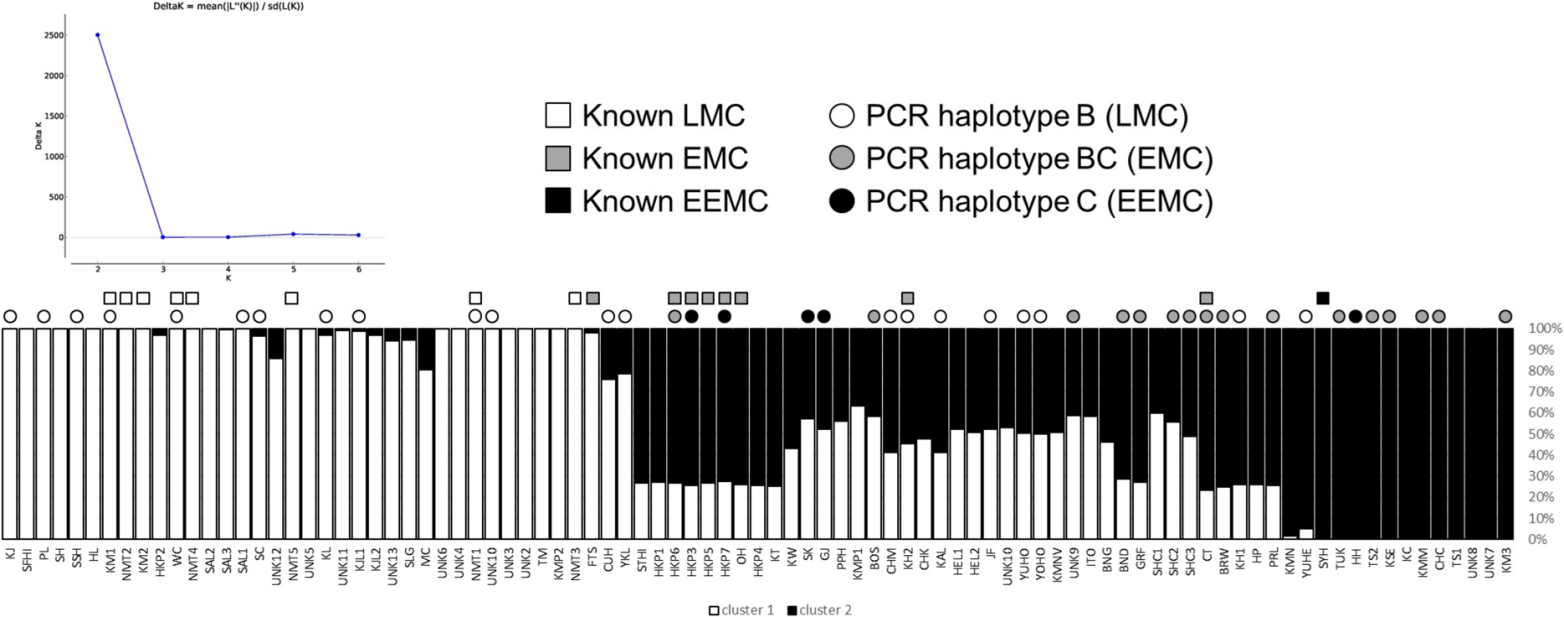
STRUCTURE analysis of 91 lychee samples. **A** the deltaK criterion for values of K (the number of clusters) from 2 to 6. **B** STRUCTURE results, samples are listed from left to right in order of appearance on the NJ tree (Fig. 2). Each bar represents an individual with white and black representing membership to clusters 1 and 2, respectively

The NJ phylogenetic tree (Fig. 2) demonstrates two divergent groups with a range of intermediates in between. The structure analysis supports this; the analysis identified that two clusters (i.e., K = 2) best represented the clustering relationships among the accessions (Fig. 3a). Thirty-six accessions have > 80% membership in cluster 1 and 14 accessions have > 80% membership in cluster 2 with the remaining 41 showing less than 80% membership into one of the clusters.

The degree of admixture was highly variable; however, the admixed individuals did not form a gradation from cluster 1 to cluster 2 and instead a substantial proportion of admixed individuals were very close to the 25/75 or 50/50 expectation for a BC1 or F1 individual (Fig. 3b). Thirteen of the 41 individuals had 25/75 (± 2%) admixture and five had 50/50 (± 2%) admixture. Together our genetic analyses confirm the previous finding that there are two subgroups of lychee cultivars, with hybrids between the groups being prevalent and potentially many of these are early generation hybrids.

Accessions of Kwai Mi (KM [except for KM3 which does not cluster with the other KM accessions, see below]), No Mai Tsze (NMT) and Wai Chee (WC), previously known LMCs, had > 99% membership into cluster 1 (Fig. 3b; white bars). The single previously known EEMC (SYH, Sam Yu Hung) had 100% membership into cluster 2 (Fig. 3b; black bars). The PCR assay of 42 accessions identified 21 homozygous for the insertion (haplotype B; LMC) and 5 homozygous for the deletion (haplotype C; EEMC). Most of the individuals with haplotype B are indeed individuals in cluster 1 whereas the five haplotype C individuals are found in the admixed (n = 4) and cluster 2 (n = 1) group. Individuals with a heterozygous genotype (n = 16) are also found in the admixed and cluster 2 groups. It would appear that cluster 1 represents LMC individuals and cluster 2 has a mixture of EMC and EEMC individuals, according to their genotype.

### Synonymous and homonymous cultivars

The phylogenetic and structure analyses allow for visualisation of potential instances of misidentified samples, for example individuals of Kwai Mi (KM), Kwai Mi Pink (KMP) and No Mai Tsze (NMT) are scattered across the phylogenetic tree (Fig. 2). To provide an objective genetic distance cut-off for identical vs non-identical samples we first identified pairs of identically named individuals (or known synonymous cultivars) that were sister in the phylogenetic tree, however only three pairs were revealed, despite nine cultivars being represented by more than one (up to seven) individual(s). These three pairs were Shan Chi (SHC2/3; group 1 in Fig. 2; genetic distance 0.0651), Heung Lai (HEL1/2; group 2 in Fig. 2; 0.0442) and the synonymous cultivars (Anuntalabhochai et al., 2000; Menzel 2002) O-Hia and Hak ip (OH/HKP7; group 3 in Figure; 0.0413). Residual distance between identical cultivars is most likely due to miss-calling of heterozygotes as homozygotes from low sequencing read-depth, as is typical in high-multiplexing, sequence-based genotyping methods, as well as the potential for minor variants originating and being propagated clonally.

If we expand this to include pairs of accessions of the same cultivar grouped with other samples in tight phylogenetic groups then this adds another three groups: group 3b (i.e., expanding group 3): HKP1/3/4/5/6/7, OH and STHI (genetic distance 0.0376—0.0564), group 4: KJL1/2 and UNK11 (0.0310—0.0482), and group 5: SAL1/2/3, NMT4 and WC (0.0316—0.0482) (Fig. 2, blue groups). Overall, the maximum distance among individuals within those five groups is 0.0651, which was from one of the varietal pairs (SCHI1/2).

We applied this value as a cut-off for identifying identical samples, and In doing so, eight further groups (i.e., in addition to those above) are identified (Fig. 2, green groups). Details on the eight new groups are as follows. Group 6 is a heterogenous group of Hwai Lai (HL), Shang Shu Hwai (SSH), Kim Jin (KJ), Pak Larp (PL), Sun Fuon Hak Ip (SFHI), Sun Hing (SH) and one of three Kwai Mi (KM1). Group 7 pairs UNK12 and No Mai Tsze 5. Group 8 combines five unknown samples (UNK1/2/3/4/6) with No Mai Tsze 1. Group 9 combines Tim Nagn, one Kwai Mi Pink (KMP2) and one No Mai Tsze (NMT3). Group 10 pairs unknown 10 with Jun Fuon. Group 11 pairs unknown 9 with Ito. Group 12 combines Brewster (BRW), Hong Pea (HP), Peerless (PRL) and one Khom individual (KH1). Group 13 contains a range of cultivars, namely Tai So (TS), Chacapot (CHC), Kau Ching (KC), Kwai Mei – Mauritius (KMM), Kao Shung Early (KSE), one Kwai Mi (KM3) and two Unknowns (7 and 8). Finally, based on the cut-off, Kwang Tung (KT) is added to group 3b (HKP1/3/4/5/6/7, OH and STHI).

In sum, for four of the cultivars with multiple accessions [Heung Lai (HEL), Kai Ju Lai (KJL), Tai So (TS) and Salathiel (SAL)], all members form a group with a genetic distance less than the cut-off. In addition, except for one individual, Hak Ip (HKP) and Shan Chi (SHC) form clusters. Several other cultivars appear to be homonyms, for example Khom (KH), Kwai Mi (KM), Kwai Mi Pink (KMP) and No Mai Tsze (NMT). The five No Mai Tsze individuals do not group with each other at all and are found in different clades of the phylogeny (Fig. 2, black circles). Based on our cut-off, 55 of the 91 trees analysed were clones, in groups of 2–9 individuals. Overall, there were 13 groups of clones (Table 2) and 36 non-clonal individuals (i.e., in total 49 different accessions).

**Table 2:** Summary of the 13 groups of clonal individuals

Group	Size	Individuals*
1	2	SHC2, SHC3
2	2	HEL1, HEL2
3b	9	OH, HKP1, HKP3, HKP4, HKP5, HKP6, HKP7, STHI, KT
4	3	KJL1, KJL2, UNK11
5	5	SAL1, SAL2, SAL3, NMT4, WC
6	7	HL, SSH, KJ, PL, SFHI, SH, KM1
7	2	UNK12, NMT5
8	6	UNK1, UNK2, UNK3, UNK4, UNK6, NMT1
9	3	TM, KMP2, NMT3
10	2	UNK10, JF
11	2	UNK9, ITO
12	4	BRW, HP, PRL, KH1
13	8	TS2, CHC, KC, KMM, KSE, KM3, UNK7, UNK8

^*^Abbreviations are given in online resource 1

We can suggest whether the samples without varietal names (‘unknown’) are members of existing cultivars or unique. Unknowns 9, 10 and 11 appear to represent Ito (group 11), Jun Fong (group 10) and Kai Ju Lai (group 4), respectively. Unknowns 1, 2, 3, 4, and 6 group with No Mai Tsze 1 and unknown 12 groups with No Mai Tsze 5; however, given the paraphyly of No Mai Tsze it would be premature to conclude that either of these groups is the ‘real’ No Mai Tsze. Unknowns 7 and 8 group with several other named cultivars in group 13, which themselves require some reassessment. The final two unknown accessions without cultivar names (5 and 13) are not found to be closely related to other accessions.

### A core SNP set

CoreSNP identified just 12 SNP markers that could differentiate all samples in the study (Table 3; Online Resource 5).

**Table 3:** The results of the CoreSNP analysis

SNP_ID	CHROM	POSITION	MAF	PIC	Reference allele	Alternate allele
Chr1_8010712T	1	8,010,712	0.4368	0.371	C	T
Chr2_27283681T	2	27,283,681	0.4828	0.375	C	T
Chr6_5368453C	6	5,368,453	0.4500	0.372	T	C
Chr6_8298067G	6	8,298,067	0.4540	0.373	A	G
Chr8_3936102A	8	3,936,102	0.3678	0.357	T	A
Chr8_6936632T	8	6,936,632	0.5000	0.375	C	T
Chr8_14412695C	8	14,412,695	0.4719	0.374	T	C
Chr14_3021566C	14	3,021,566	0.3820	0.361	T	C
Chr14_16181907C	14	16,181,907	0.4885	0.375	T	C
Chr14_20281020A	14	20,281,020	0.4828	0.375	G	A
Chr15_21456669A	15	21,456,669	0.4943	0.375	G	A
Chr15_23479714T	15	23,479,714	0.4713	0.374	G	T

12 SNP markers were identified that could differentiate the samples. *MAF* minor allele frequency, *PIC* polymorphic information content. Flanking sequences are available in Online Resource 5

## Discussion

We used ca. 10,000 SNPs to analyse the relationships between 91 lychee individuals from the US Department of Agriculture (USDA) lychee collection growing in Hawai’i. Overall, we find strong support for previous work wherein the cultivars comprise two genetically divergent groups with several admixed individuals in-between. There are no obvious morphological differences between the two main groups, however flowering on Hawai’i is too sporadic to have complete data on flowers or fruits.

We would not expect clonal lineages to be 100% identical using this highly multiplexed approach, where miscalling of heterozygotes, sequencing errors and inconsistencies in the restriction enzyme digestion can all occur, therefore a cutoff of 100% is not appropriate. The approach we employed to identify synonymous cultivarsused the genetic distance between known pairs as the cut-off for identical vs not identical.

Based on our grouping of individuals to identify synonymous cultivars we suggest there are several synonymous cultivars in the collection; 13 groups of between 2 and 9 trees were suggested to be clones (Table 2). Some of these were suggested previously, and/or could be suggested based on similar names. For example, Hak Ip and O-Hia were previously shown to be synonymous (Anuntalabhochai et al. 2002; Menzel 2002), and we find them as sister accessions. Also in this group (group 3b; Fig. 2), we find Shui Tung Hak ip and therefore the name suggests it is related to Hak Ip. No Mai Tsze (Chinese) had been previously suggested to be identical to Wai Chee (Thai) and Salathiel (Australia) (Menzel 2002) and our data support this (group 5; Fig. 1), however given that only one of the five No Mai Tsze group here, and the other four are found in four distinct groups, whether this is the genuine No Mai Tsze remains to be assessed. Group 13 contains a range of cultivars, namely Tai So (TS), Chacapot (CHC), Kau Ching (KC), Kwai Mei – Mauritius (KMM), Kao Shung Early (KSE), one Kwai Mi (KM3) and two Unknowns (7 and 8). Tai So is the Australian name for Mauritius from the Philippines and Hong Huay from Thailand (Menzel 2002). The similarity between Tai So and Mauritius is therefore backed up, but Hong Huay (if the sample Hohng Hooway [HH] is this) is slightly more distantly related (genetic distance ca. 0.11). Aradhya et al. (1995) suggested that Kwai Mi (KM) and Kau Ching (KCG) are synonymous with TS and KMM, which our data backs up. This would suggest that KM3 is a genuine KM because it groups with TS and KMM in group 13, and KM1 and KM2 are not.

Several genotypes known as Hak Ip have been shown to be genetically different (Degani et al. 1995) and we found HKP2 to be divergent from the other six individuals. ‘Hak Ip’ has been introduced to Hawaii on three separate occasions from Israel, South Africa, and Florida (Goren and Gazit 1994, cited in Degani et al. 1995).

Groff (GRF) and Kaimana (KMN) were developed independently from Hak Ip seedlings (Chia et al. 1997). GRF and KMN do not pair with each other nor group with any other accessions which could indicate their origin through hybridisation between cultivars. Records at the USDA suggest that Chan Tsee is a modified name for Chen Zi and this is thought to be identical to Brewster, which appears to be correct from our work (group 12). Sample UNK12 was from Waiakea arboretum on Hawai’i and had been thought to be related to NMT which it does appear to be, however again because NMT is so widely distributed across the tree, therefore continuing to use this name might need reconsideration. UNK9 is apparently a selection from a Kaimana (KMN) seedling, although it appears more closely related to Ito, however sister to these is a cultivar called Kaimana Variant.

Aradhya et al. (1995) states that Bosworth-3 (BOS) was created from a cutting of Kwai Mi Pink (KMP) and so should be a clone, Fig. 2 supports BOS and KMP1 are closely related but did not meet our criterion for being included as synonymous. Given that KMP1 and KMP2 are very distantly related, we can assume that KMP2 is a misidentified sample because of KMP1’s close relation to BOS.

Going forward, future research can use our panel of core SNP markers with high power to differentiate cultivated lychees to expedite investigations into other cultivars. A previous study that included wild lychees found that 14 SNPs were required to differentiate all the material (Liu et al. 2015), therefore only 12 seems to be appropriate for when just cultivated material is being used.

### Fruit maturation

Fruit maturation time has been used as the primary determinant of genetic relationships within lychee and is a valuable characteristic (Menzel 2002). A previous genomic analysis (Hu et al. 2022) found that the two main groups were extremely early (EEMC) or late maturing cultivars (LMC) with intermediates being early (EMC). Hu et al. (2022) showed complete distinction between LMC and EEMC genotypes for a 3.7-kb presence/absence variant downstream of a CONSTANS-like gene (COL307) gene. Our work demonstrates that cluster 1 samples are LMC based on prior knowledge of maturity times, and these are all homozygous for haplotype B, matching the Hu et al. (2022) results. In contrast, while the cluster 2 individuals are probably EEMC (based on one known maturity time), these comprise a mixture of haplotype C and heterozygous (BC) individuals. Given the significant admixture we identified for several individuals, it appears that hybridisation has blurred the distinction between LMC and EEMC cultivars at the *COL307* locus.

## Conclusion

The identification of synonymous cultivars greatly improves our understanding of genetic relationships within lychee. This will lead to better organised germplasm management and improvement in the performance of conservation and breeding programmes (Tran et al. 2019). However, if two synonymous cultivars are cultivated in different locations and as such produce different morphologies to each other, they should be kept as distinct on commercial markets because they offer variation to the consumer. To fix future naming issues, we suggest that names which indicate their genetic synonymity but also their morphological difference, for example, ‘No Mai Tsze’ and ‘No Mai Tsze Red’. This system allows for easy identification of synonymous cultivars for records and management, whilst also highlighting variation between different phenotypes for consumers and growers.

Finally, only 69 out of several hundred named cultivars were included in this study, with a further 150 analysed in other publications (Liu et al. 2015; Tran et al. 2019; Hu et al. 2022), meaning that many cultivars that exist have not been analysed. Most cultivars that have not been investigated are, understandably, less important cultivars, although analysing these may help identify genetic basis of adaptive, agronomic or commercial phenotypes (Della Coletta et al. 2021; Chapman et al. 2022), feeding into lychee breeding programmes. Given our finding that the number of named cultivars appears an overestimate of the number of genetically distinct genotypes, further investigations analysing the remaining cultivars are needed to ensure that genetically divergent cultivars are identified and preserved. The diagnostic SNPs we identified that, when used as a panel, can differentiate trees in the USDA collection could be developed into an assay to genotype further trees, both cultivated and wild.

## Supplementary Information

Below is the link to the electronic supplementary material.

Supplementary file1 (XLSX 12 KB)

Supplementary file2 (PPTX 329 KB)

## Data Availability

Raw reads for the lychee samples have been uploaded to NCBI-SRA (accession number PRJNA1230099).

## References

[CR1] Anuntalabhochai S, Chundet R, Chiangda J, Apavatjrut P (2002) Genetic diversity within lychee (Litchi chinensis sonn.) Based on RAPD analysis. Int Soc Horticultural Sci (ISHS). 10.17660/ActaHortic.2002.575.27

[CR2] Aradhya MK, Zee FT, Manshardt RM (1995) Isozyme variation in lychee (*Litchi chinensis* Sonn.). Sci Hortic 63:21–35. 10.1016/0304-4238(95)00788-U

[CR3] Azam-Ali SN (2010) Fitting underutilised crops within research-poor environments: Lessons and approaches. South African J Plant and Soil 27:293–298. 10.1080/02571862.2010.10639997

[CR4] Baric S, Storti A, Hofer M, Dalla Via J (2009) Molecular genetic characteristion of apple cultivars from different germplasm collections. Int Soc Horticultural Sci (ISHS). 10.17660/ActaHortic.2009.817.37

[CR5] Cabezas JA, Ibáñez J, Lijavetzky D, Vélez D, Bravo G, Rodríguez V, Carreño I, Jermakow AM, Carreño J, Ruiz-García L et al (2011) A 48 SNP set for grapevine cultivar identification. BMC Plant Biol 11:153. 10.1186/1471-2229-11-153 22060012 10.1186/1471-2229-11-153 PMC3221639

[CR6] Cabral TA, CardosoPinheiro-Sant’Ana LdMHM (2014) Chemical composition, vitamins and minerals of a new cultivar of lychee (*Litchi chinensis* cv. Tailandes) grown in Brazil. Fruits 69:425–434. 10.1051/fruits/2014031

[CR7] Carvajal-Yepes M, Ospina JA, Aranzales E, Velez-Tobon M, Correa Abondano M, Manrique-Carpintero NC, Wenzl P (2023) Identifying genetically redundant accessions in the world’s largest cassava collection. Front Plant Sci 14:1338377. 10.3389/fpls.2023.1338377 38304449 10.3389/fpls.2023.1338377 PMC10830726

[CR8] Chapman MA (2019) Optimizing depth and type of high-throughput sequencing data for microsatellite discovery. Applications in Plant Sciences 7:e11298. 10.1002/aps3.11298 31832281 10.1002/aps3.11298 PMC6858294

[CR9] Chapman MA, He Y, Zhou M (2022) Beyond a reference genome: pangenomes and population genomics of underutilized and orphan crops for future food and nutrition security. New Phytol 234:1583–1597. 10.1111/nph.18021 35318683 10.1111/nph.18021 PMC9994440

[CR10] Chapman KR (1984) Sapindaceae. In: Tropical Fruit Tree Fruits for Australia. Queensland Department of Primary Industries. pp. 189–191.

[CR11] Chapman MA, ed. (2022) Underutilised Crop Genomes. Springer Nature, Switzerland. 10.1007/978-3-031-00848-1

[CR12] Chia CL, Hamilton RA, Exvans DO (1997). Horticultural Commodity Fact Sheet no. 1 Lychee. in Hawaii Cooperative Extension Service. Avaialable at http://hdl.handle.net/10125/12647

[CR13] Danecek P, Auton A, Abecasis G, Albers CA, Banks E, DePristo MA, Handsaker RE, Lunter G, Marth GT, Sherry ST et al (2011) The variant call format and VCFtools. Bioinformatics 27:2156–2158. 10.1093/bioinformatics/btr330 21653522 10.1093/bioinformatics/btr330 PMC3137218

[CR14] Degani C, Beiles A, El-Batsri R, Goren M, Gazit S (1995) Identifying Lychee Cultivars by Isozyme Analysis. J Am Soc Hort Sci. 120:307–312. 10.21273/JASHS.120.2.307

[CR15] Della Coletta R, Qiu Y, Ou S, Hufford MB, Hirsch CN (2021) How the pan-genome is changing crop genomics and improvement. Genome Biol 22:3. 10.1186/s13059-020-02224-8 33397434 10.1186/s13059-020-02224-8 PMC7780660

[CR16] Dou T, Wang C, Ma Y, Chen Z, Zhang J, Guo G (2023) CoreSNP: an efficient pipeline for core marker profile selection from genome-wide SNP datasets in crops. BMC Plant Biol 23:580. 10.1186/s12870-023-04609-w 37986037 10.1186/s12870-023-04609-w PMC10662547

[CR17] Doyle JJ, Doyle JL (1990) Isolation of plant DNA from fresh tissue. Focus 12:13–15

[CR18] Evanno G, Regnaut S, Goudet J (2005) Detecting the number of clusters of individuals using the software STRUCTURE: a simulation study. Mol Ecol 14:2611–2620. 10.1111/j.1365-294X.2005.02553.x 15969739 10.1111/j.1365-294X.2005.02553.x

[CR19] FAO (2002). Lychee Production in the Asia-Pacific Region.Food And Agriculture Organization of The United Nations Regional Office for Asia and The Pacific.

[CR20] FAO (2010). The Second Report on the State of the World’s Plant Genetic Resources for Food and Agriculture.

[CR21] FAO (2017). The future of food and agriculture- trends and challenges., Rome, Italy.

[CR22] FAO (2020). FAOSTAT database collections Food and Agriculture Organization of the United Nations, Rome, Italy.

[CR23] Felsenstein J (2004) PHYLIP (Phylogeny Inference Package) version 3.72. Distributed by the author. Department of Genome Sciences, University of Washington, Seattle. http://evolution.gs.washington.edu/phylip.html.

[CR24] Godfray HCJ, Beddington JR, Crute IR, Haddad L, Lawrence D, Muir JF, Pretty J, Robinson S, Thomas SM, Toulmin C (2010) Food Security: The Challenge of Feeding 9 Billion People. Science 327:812–818. 10.1126/science.1185383 20110467 10.1126/science.1185383

[CR25] Goren M, Gazit S (1994) New promising lychee cultvars for Israel in 1993—Description, evaluation and recommendations. Alon haNotea 48:202–206

[CR26] Hamblin MT, Rabbi IY (2014) The effects of restriction-enzyme choice on properties of genotyping-by-sequencing libraries: a study in cassava (*Manihot esculenta*). Crop Sci 54:2603–2608. 10.2135/cropsci2014.02.0160

[CR27] Hu G, Feng J, Xiang X, Wang J, Salojärvi J, Liu C, Wu Z, Zhang J, Liang X, Jiang Z et al (2022) Two divergent haplotypes from a highly heterozygous lychee genome suggest independent domestication events for early and late-maturing cultivars. Nat Genet 54:73–83. 10.1038/s41588-021-00971-3 34980919 10.1038/s41588-021-00971-3 PMC8755541

[CR28] Ibrahim SRM, Mohamed GA (2015) *Litchi chinensis*: medicinal uses, phytochemistry, and pharmacology. J Ethnopharmacol 174:492–513 26342518 10.1016/j.jep.2015.08.054

[CR29] Kopelman NM, Mayzel J, Jakobsson M, Rosenberg NA, Mayrose I (2015) Clumpak: a program for identifying clustering modes and packaging population structure inferences across K. Mol Ecol Resour 15:1179–1191. 10.1111/1755-0998.12387 25684545 10.1111/1755-0998.12387 PMC4534335

[CR30] Lefort V, Desper R, Gascuel O (2015) FastME 2.0: a comprehensive, accurate and fast distance-based phylogeny inference program. Mol Biol Evol 32:2798–2800. 10.1093/molbev/msv150 26130081 10.1093/molbev/msv150 PMC4576710

[CR31] Letunic I, Bork P (2021) Interactive Tree Of Life (iTOL) v5: an online tool for phylogenetic tree display and annotation. Nucleic Acids Res 49:W293–W296. 10.1093/nar/gkab301 33885785 10.1093/nar/gkab301 PMC8265157

[CR32] Li H, Durbin R (2009) Fast and accurate short read alignment with Burrows-Wheeler transform. Bioinformatics 25:1754–1760. 10.1093/bioinformatics/btp324 19451168 10.1093/bioinformatics/btp324 PMC2705234

[CR33] Li H, Handsaker B, Wysoker A, Fennell T, Ruan J, Homer N, Marth G, Abecasis G, Durbin R (2009) The Sequence Alignment/Map format and SAMtools. Bioinformatics 25:2078–2079. 10.1093/bioinformatics/btp352 19505943 10.1093/bioinformatics/btp352 PMC2723002

[CR34] Liu W, Xiao Z, Bao X, Yang X, Fang J, Xiang X (2015) Identifying litchi (*Litchi chinensis* Sonn.) cultivars and their genetic relationships using single nucleotide polymorphism (SNP) markers. PLoS ONE 10:e0135390. 10.1371/journal.pone.0135390 26261993 10.1371/journal.pone.0135390 PMC4532366

[CR35] Liu W, Xiao Z, Jiang N, Fan C, Xiang X (2023) Genome-Wide development of polymorphic SNP markers and evaluation of genetic diversity of litchi (Litchi chinensis Sonn). Plants (Basel) 12:3949. 10.3390/plants12233949 38068586 10.3390/plants12233949 PMC10708096

[CR36] Mayes S, Massawe FJ, Alderson PG, Roberts JA, Azam-Ali SN, Hermann M (2012) The potential for underutilized crops to improve security of food production. J Exp Bot 63:1075–1079. 10.1093/jxb/err396 22131158 10.1093/jxb/err396

[CR37] McKenna A, Hanna M, Banks E, Sivachenko A, Cibulskis K, Kernytsky A, Garimella K, Altshuler D, Gabriel S, Daly M et al (2010) The genome analysis toolkit: a MapReduce framework for analyzing next-generation DNA sequencing data. Genome Res 20:1297–1303. 10.1101/gr.107524.110 20644199 10.1101/gr.107524.110 PMC2928508

[CR38] Menzel CM, Simpson DR (1987) Lychee nutrition: A review. Sci Hortic 31:195–224

[CR39] Menzel CM, Simpson DR (1990) Effect of temperature on growth and flowering of litchi (*Litchi chinensis* Sonn.) cultivars. J Hort Sci and Biotech 63:349–360. 10.1080/14620316.1988.11515869

[CR40] Menzel C (2002) The Lychee Crop in Asia And the Pacific. FAO Regional Office for Asia and the Pacific, Bangkok, Thailand.

[CR41] Mitra SK, Pan J (2020). Litchi and longan production and trade in the world. pp. 1–6. International Society for Horticultural Science (ISHS), Leuven, Belgium. 10.1016/0304-4238(87)90046-X

[CR42] Morton J (1987) Lychee. pp. 249–259. In: Fruits of warm climates. Julia F. Morton Miami FL.

[CR43] Nath V, Pandey SD, Pongener A, Srivastava K, Marboh ES (2018) Challenges and opportunities for improved productivity of litchi. Acta Hortic 1211:161–164. 10.17660/ActaHortic.2018.1211.22

[CR44] Pritchard JK, Stephens M, Donnelly P (2000) Inference of population structure using multilocus genotype data. Genetics 155:945–959. 10.1093/genetics/155.2.945 10835412 10.1093/genetics/155.2.945 PMC1461096

[CR45] Purcell S, Neale B, Todd-Brown K, Thomas L, Ferreira MA, Bender D, Maller J, Sklar P, de Bakker PI, Daly MJ et al (2007) PLINK: a tool set for whole-genome association and population-based linkage analyses. Am J Hum Genet 81:559–575. 10.1086/519795 17701901 10.1086/519795 PMC1950838

[CR46] Rabbi IY, Kulakow PA, Manu-Aduening JA, Dankyi AA, Asibuo JY, Parkes EY, Abdoulaye T, Girma G, Gedil MA, Ramu P et al (2015) Tracking crop varieties using genotyping-by-sequencing markers: a case study using cassava (*Manihot esculenta* Crantz). BMC Genet 16:115. 10.1186/s12863-015-0273-1 26395668 10.1186/s12863-015-0273-1 PMC4580218

[CR47] Riaz S, Boursiquot J-M, Dangl GS, Lacombe T, Laucou V, Tenscher AC, Walker MA (2013) Identification of mildew resistance in wild and cultivated Central Asian grape germplasm. BMC Plant Biol 13:149. 10.1186/1471-2229-13-149 24093598 10.1186/1471-2229-13-149 PMC3851849

[CR48] Ross-Ibarra J, Morrell PL, Gaut BS (2007) Plant domestication, a unique opportunity to identify the genetic basis of adaptation. Proc Natl Acad Sci USA 104:8641–8648. 10.1073/pnas.0700643104 17494757 10.1073/pnas.0700643104 PMC1876441

[CR49] Saitou N, Nei M (1987) The neighbor-joining method: a new method for reconstructing phylogenetic trees. Mol Biol Evol 4:406–425. 10.1093/oxfordjournals.molbev.a040454 3447015 10.1093/oxfordjournals.molbev.a040454

[CR50] Song L, Cao B, Zhang Y, Meinhardt LW, Zhang D (2021) Mining single nucleotide polymorphism (SNP) markers for accurate genotype identification and diversity analysis of chinese jujube (Ziziphus jujuba Mill.) Germplasm. In Agronomy. 10.3390/agronomy11112303

[CR51] Sun X, Liu D, Zhang X, Li W, Liu H, Hong W, Jiang C, Guan N, Ma C, Zeng H et al (2013) SLAF-seq: an efficient method of large-scale De Novo SNP discovery and genotyping using high-throughput sequencing. PLoS ONE 8:e58700. 10.1371/journal.pone.0058700 23527008 10.1371/journal.pone.0058700 PMC3602454

[CR52] Sun W, Shahrajabian MH, Shen H, Cheng Q (2021) Lychee (*Litchi chinensis* Sonn.), the king of fruits, with both traditional and modern pharmacological health benefits. Pharmacognosy Communications 11:22–25. 10.5530/pc.2021.1.5

[CR53] Tran H, Kanzaki S, Triest L, Hormaza I, Kuk NJ, Ming R, Bousquet J, Khasa D, Van Damme P (2019) Analysis of genetic diversity of lychee (*Litchi chinensis* Sonn.) and wild forest relatives in the Sapindaceae from Vietnam using microsatellites. Genet Resour Crop Evol 66:1653–1669. 10.1007/s10722-019-00837-y

[CR54] USDA (2018) Litchi Collection. https://www.ars.usda.gov/pacific-west-area/hilo-hi/daniel-k-inouye-us-pacific-basin-agricultural-research-center/tropical-plant-genetic-resources-and-disease-research/docs/litchi-collection/.

[CR55] Villano C, Procino S, Blaiotta G, Carputo D, D’Agostino N, Di Serio E, Fanelli V, La Notte P, Miazzi MM, Montemurro C et al (2023) Genetic diversity and signature of divergence in the genome of grapevine clones of Southern Italy varieties. Front Plant Sci 14:1201287. 10.3389/fpls.2023.1201287 37771498 10.3389/fpls.2023.1201287 PMC10525710

[CR56] Wall MM (2006) Ascorbic acid and mineral composition of longan (Dimocarpus longan), lychee (*Litchi chinensis*) and rambutan (*Nephelium lappaceum*) cultivars grown in Hawaii. J Food Compos Anal 19:655–663. 10.1016/j.jfca.2005.12.001

[CR57] WHO (2021). Malnutrition. Available at https://www.who.int/news-room/fact-sheets/detail/malnutrition.

[CR58] Yang D-J, Chang Y-Z, Chen Y-C, Liu S-C, Hsu C-H, Lin J-T (2012) Antioxidant effect and active components of litchi (*Litchi chinensis* Sonn.) flower. Food Chem Toxicol 50:3056–3061. 10.1016/j.fct.2012.06.011 22721981 10.1016/j.fct.2012.06.011

[CR59] Yee W (1976) The lychee in Hawaii. University of Hawaii at Manoa.

[CR60] Zhang D, Vega FE, Solano W, Su F, Infante F, Meinhardt LW (2021) Selecting a core set of nuclear SNP markers for molecular characterization of Arabica coffee (*Coffea arabica* L.) genetic resources. Conserv Genet Resour 13:329–335. 10.1007/s12686-021-01201-y

[CR61] Zhao L, Wang K, Wang K, Zhu J, Hu Z (2020) Nutrient components, health benefits, and safety of litchi (*Litchi chinensis Sonn*.): A review. Compr Rev Food Sci Food Saf 19:2139–2163. 10.1111/1541-4337.12590 33337091 10.1111/1541-4337.12590

