# Supplementary material for: Genetic variation and synonymous cultivars in the USDA lychee (Litchi chinensis Sonn.) collection assessed using genome-wide SNPs
Source: Genet Resour Crop Evol. 2025 Mar 18;72(6):7373–86. doi: 10.1007/s10722-025-02406-y (PMC12317860; doi:10.1007/s10722-025-02406-y)
Supplement: Supplementary file 2 — Supplementary file2 (PPTX 329 KB) [file 10722_2025_2406_MOESM2_ESM.pptx]

## Slide 1
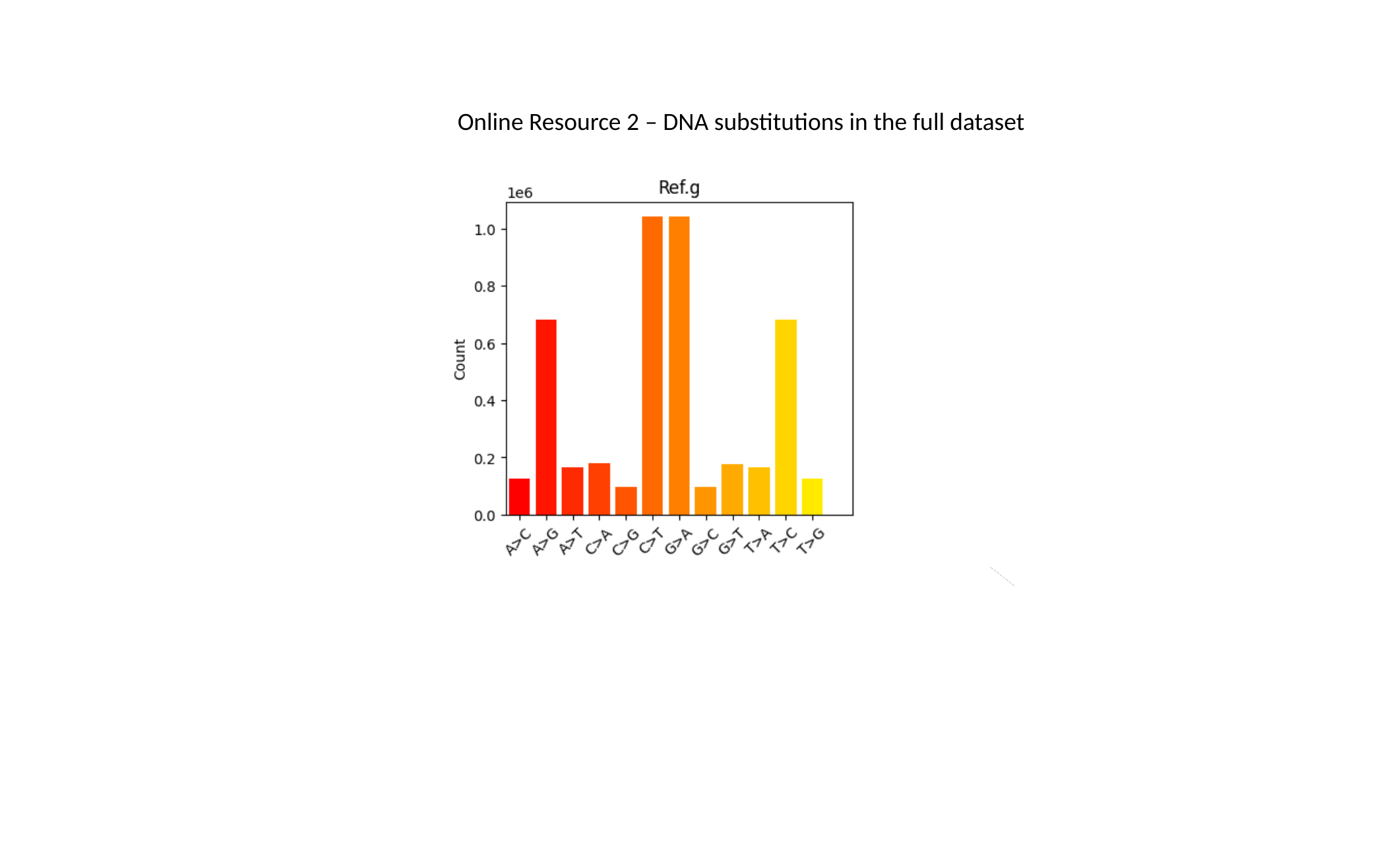

Online Resource 2 – DNA substitutions in the full dataset

## Slide 2
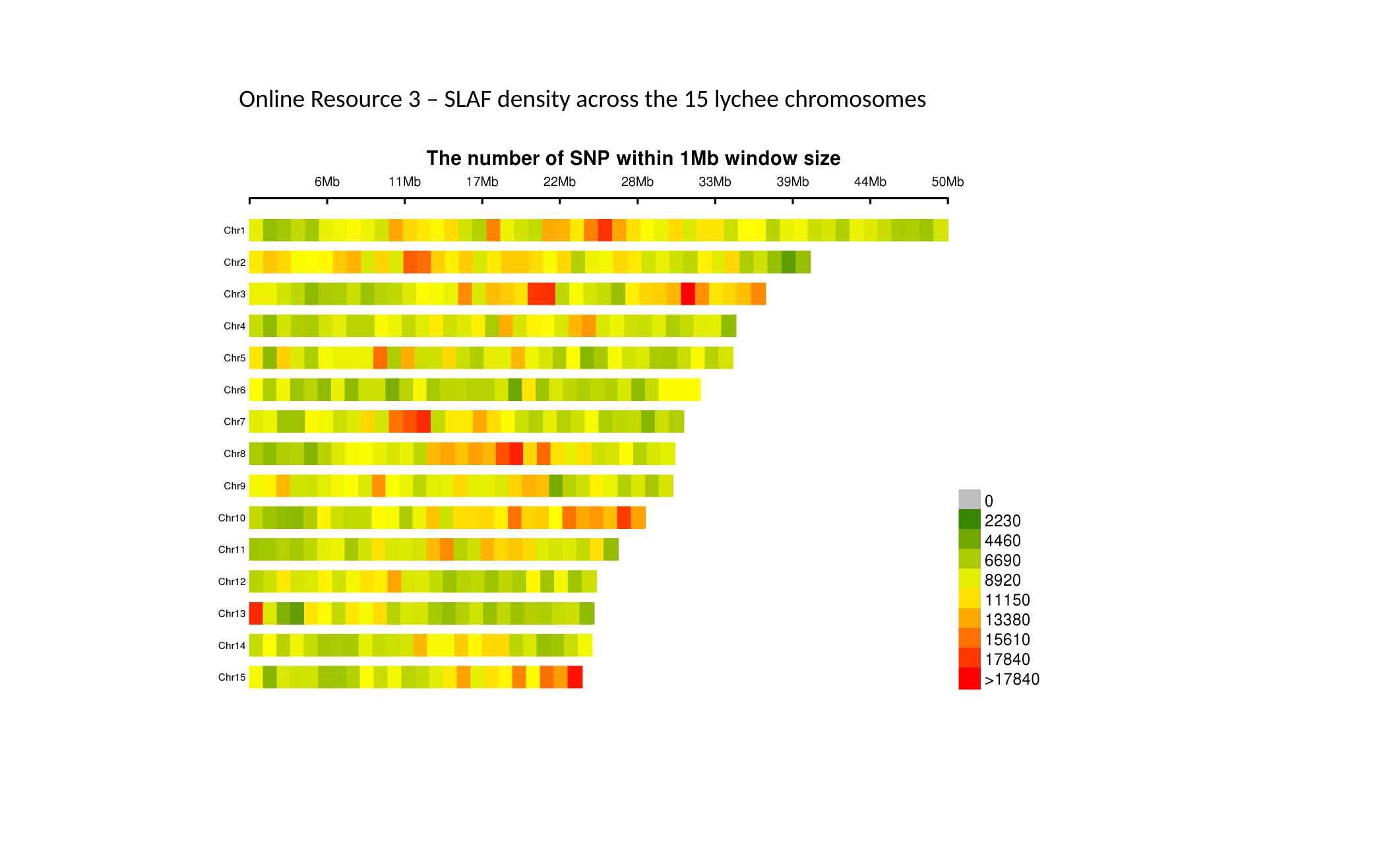

Online Resource 3 – SLAF density across the 15 lychee chromosomes

## Slide 3
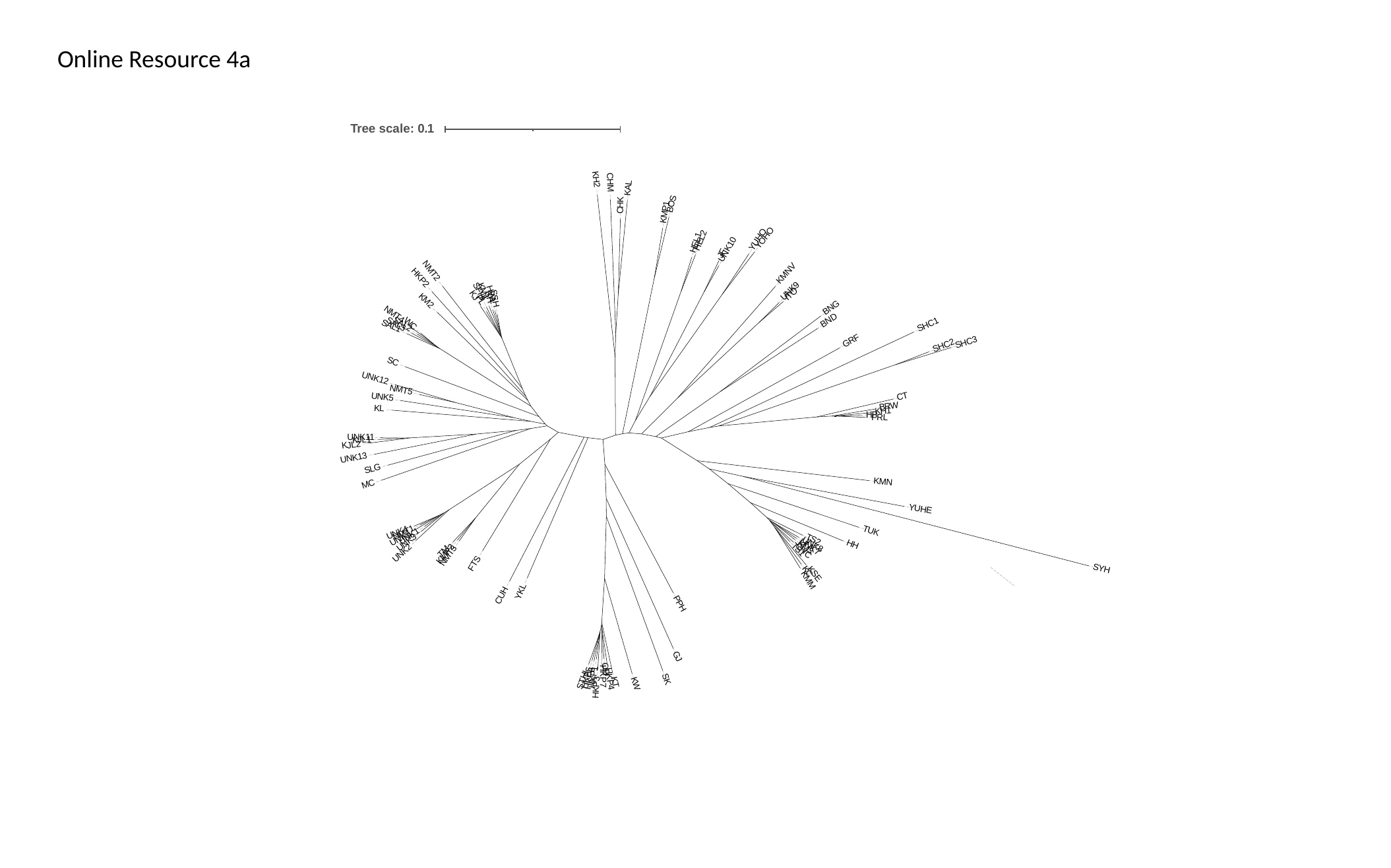

Online Resource 4a
Tree scale: 0.1
KH2
CHM
KAL
BOS
CHK
KMP1
YOHO
YUHO
HEL2
HEL1
UNK10
JF
NMT2
KMNV
HKP2
UNK9
KM1
SFHI
ITO
HWL
KJ
SH
SSH
PL
KM2
BNG
NMT4
BND
SHC1
WC
SAL3
SAL2
SAL1
GRF
SHC3
SHC2
SC
UNK12
NMT5
CT
UNK5
BRW
KL
KH1
HP
PRL
UNK11
KJL1
KJL2
UNK13
SLG
KMN
MC
YUHE
TUK
UNK4
NMT1
UNK1
UNK6
TS2
UNK3
HH
UNK8
UNK7
KM3
TM
TS1
CHC
UNK2
KMP2
NMT3
FTS
SYH
KC
KSE
KMM
YKL
CUH
PPH
GJ
HKP7
OH
HKP1
HKP5
HKP6
STHI
HKP4
SK
KT
KW
HKP3

## Slide 4
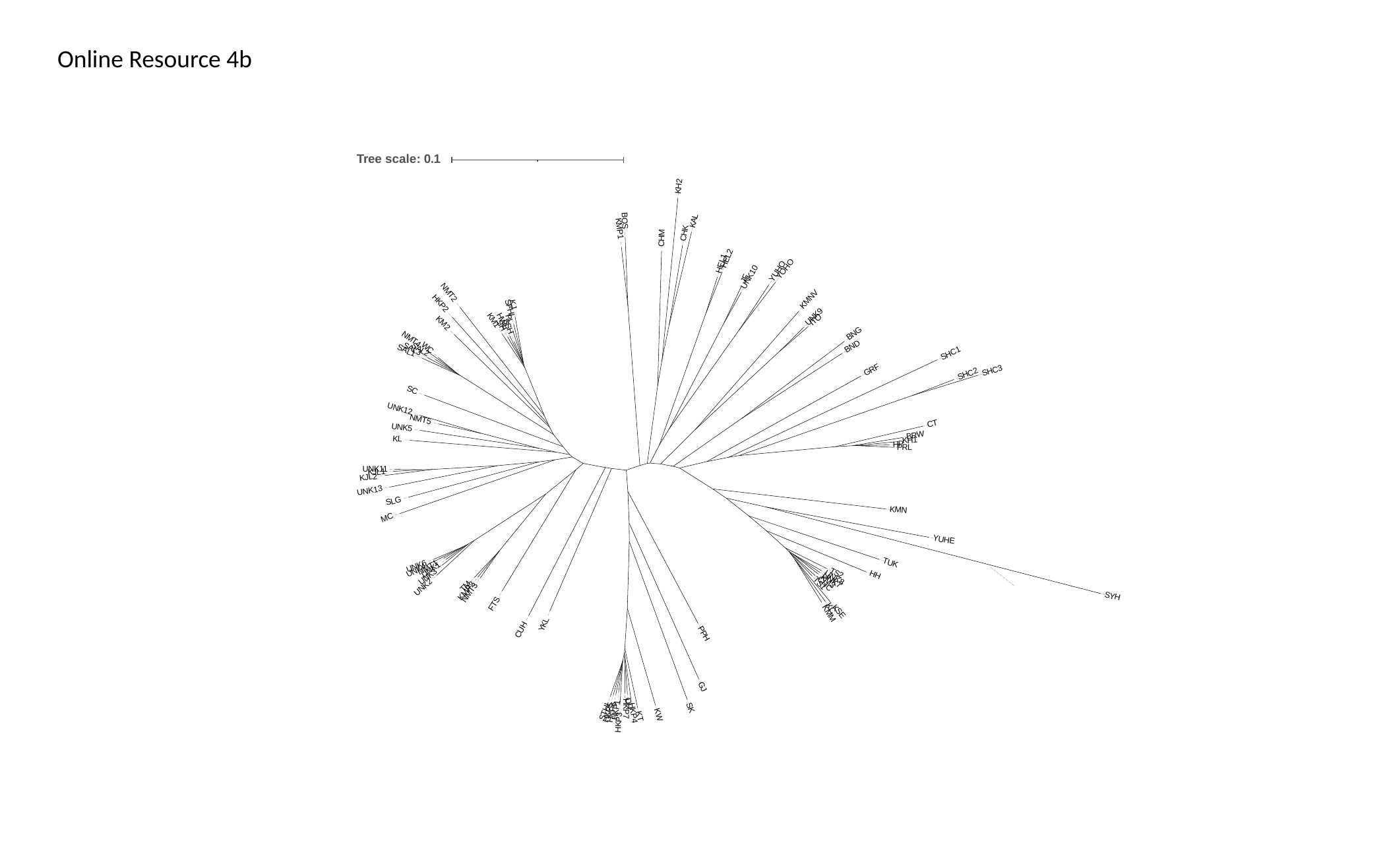

Online Resource 4b
Tree scale: 0.1
KH2
KAL
BOS
KMP1
CHK
CHM
HEL2
HEL1
YOHO
YUHO
UNK10
JF
NMT2
KMNV
HKP2
KJ
SFHI
UNK9
ITO
PL
KM1
HWL
KM2
SH
SSH
BNG
NMT4
BND
WC
SAL3
SAL2
SAL1
SHC1
GRF
SHC3
SHC2
SC
UNK12
NMT5
CT
UNK5
BRW
KL
KH1
HP
PRL
UNK11
KJL1
KJL2
UNK13
SLG
KMN
MC
YUHE
TUK
UNK6
NMT1
UNK1
UNK4
TS2
UNK3
HH
UNK8
UNK7
KM3
TS1
CHC
TM
UNK2
KMP2
NMT3
SYH
FTS
KC
KSE
KMM
YKL
CUH
PPH
GJ
HKP7
OH
HKP1
SK
STHI
HKP6
HKP5
HKP4
KW
KT
HKP3

## Slide 5
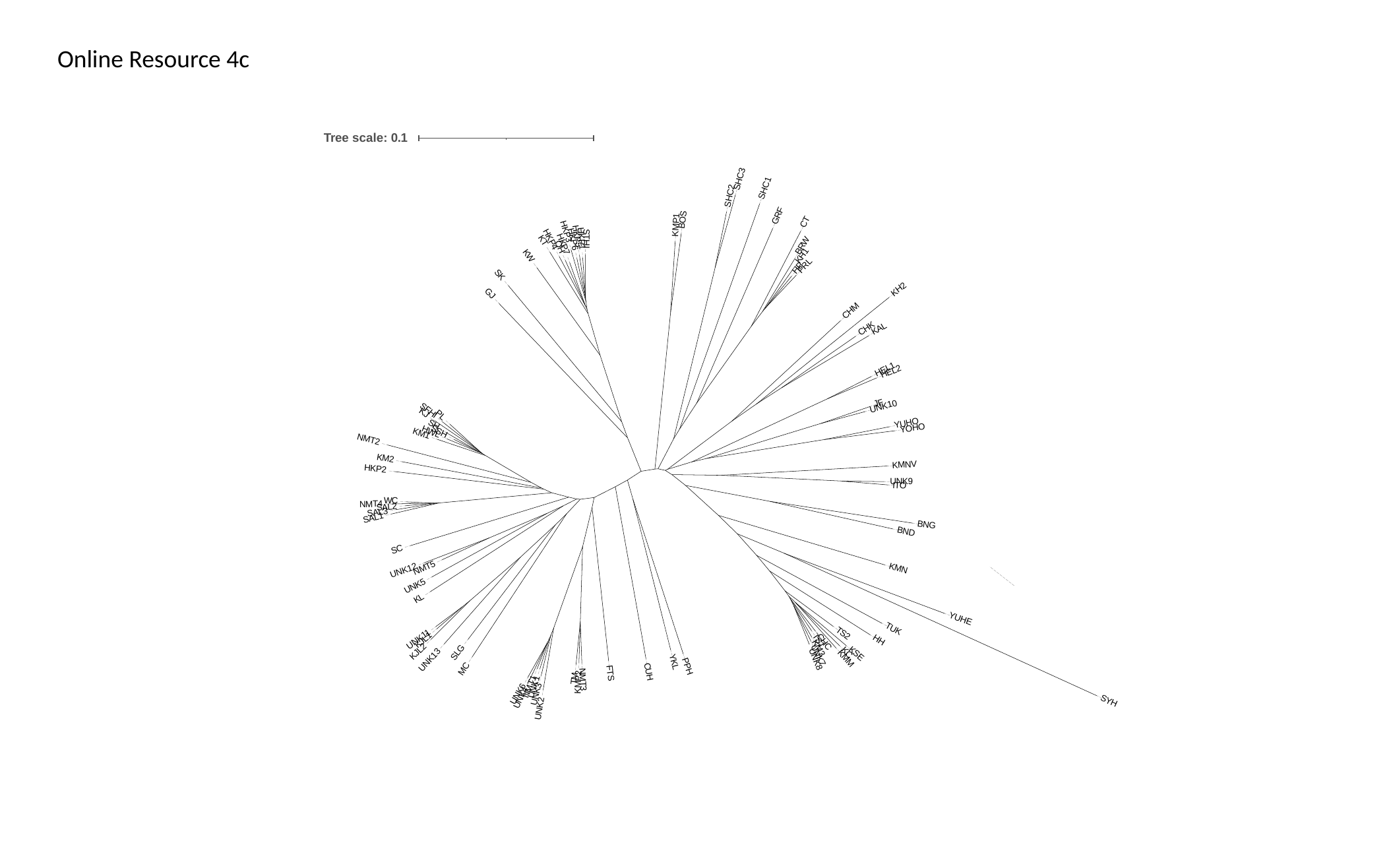

Online Resource 4c
Tree scale: 0.1
SHC3
SHC1
SHC2
GRF
BOS
CT
KMP1
STHI
HKP3
HKP5
HKP1
HKP6
HKP4
KT
BRW
HKP7
OH
KH1
KW
PRL
HP
SK
KH2
GJ
CHM
CHK
KAL
HEL1
HEL2
JF
UNK10
SFHI
KJ
PL
YUHO
SH
YOHO
SSH
HWL
KM1
NMT2
KM2
KMNV
HKP2
UNK9
ITO
WC
NMT4
SAL2
SAL3
SAL1
BNG
BND
SC
NMT5
KMN
UNK12
UNK5
KL
YUHE
TUK
TS2
UNK11
KJL1
HH
TS1
CHC
KM3
KJL2
SLG
KC
KSE
UNK7
UNK13
KMM
UNK8
YKL
MC
PPH
CUH
FTS
TM
KMP2
NMT3
NMT1
UNK1
UNK3
UNK6
UNK4
SYH
UNK2

## Slide 6
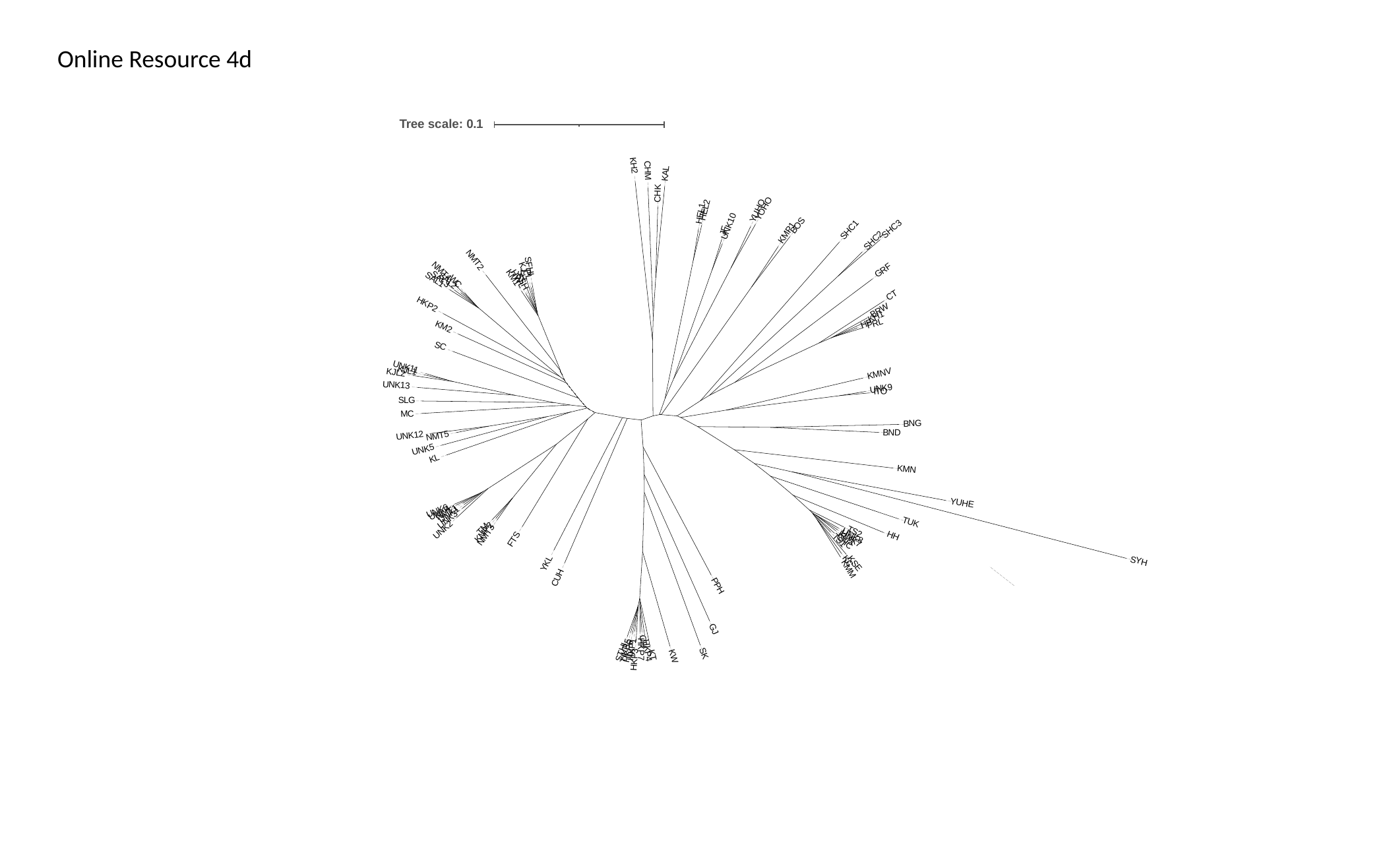

Online Resource 4d
Tree scale: 0.1
KH2
KAL
CHM
CHK
YOHO
HEL2
YUHO
HEL1
BOS
UNK10
SHC3
SHC1
JF
KMP1
SHC2
NMT2
SFHI
KJ
GRF
NMT4
PL
SH
KM1
HWL
SAL3
SAL1
SAL2
WC
SSH
CT
HKP2
BRW
KH1
HP
PRL
KM2
SC
UNK11
KJL1
KJL2
KMNV
UNK13
UNK9
ITO
SLG
MC
BNG
BND
UNK12
NMT5
UNK5
KL
KMN
YUHE
UNK6
NMT1
UNK4
UNK1
UNK3
TUK
TM
UNK2
KMP2
TS2
NMT3
UNK8
HH
FTS
KM3
UNK7
TS1
CHC
SYH
YKL
KC
KSE
KMM
CUH
PPH
GJ
HKP7
OH
HKP1
HKP5
HKP6
STHI
HKP4
SK
KT
KW
HKP3

## Slide 7
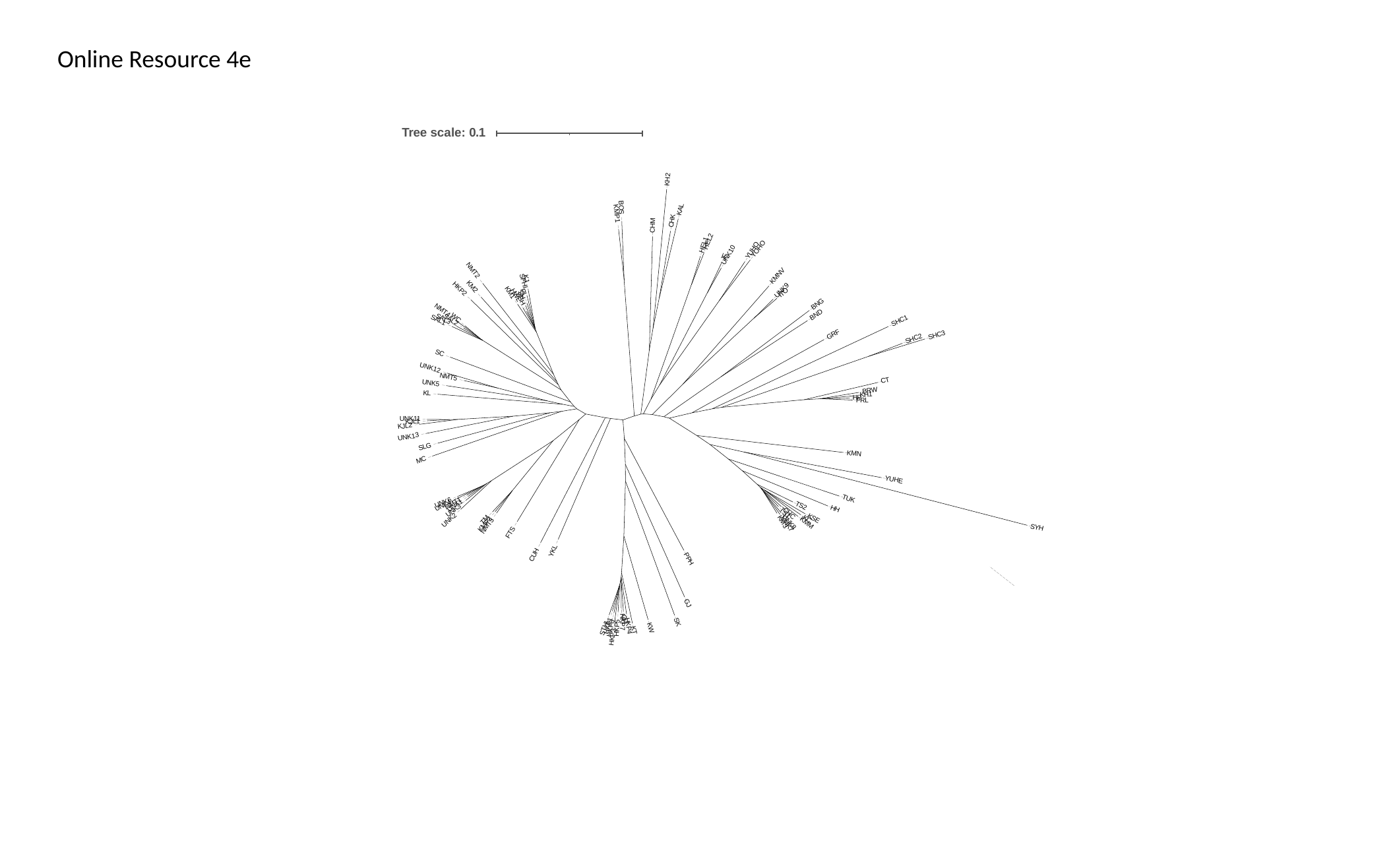

Online Resource 4e
Tree scale: 0.1
KH2
KAL
BOS
KMP1
CHK
CHM
HEL2
HEL1
YOHO
YUHO
UNK10
JF
NMT2
KMNV
KJ
SFHI
KM2
UNK9
HKP2
ITO
KM1
PL
SH
HWL
SSH
BNG
NMT4
BND
WC
SAL3
SHC1
SAL2
SAL1
GRF
SHC3
SHC2
SC
UNK12
NMT5
CT
UNK5
BRW
KL
KH1
HP
PRL
UNK11
KJL1
KJL2
UNK13
SLG
KMN
MC
YUHE
TUK
UNK6
NMT1
UNK1
UNK4
TS2
UNK3
HH
TS1
CHC
TM
UNK2
KSE
KC
KMP2
KM3
UNK8
KMM
NMT3
UNK7
SYH
FTS
YKL
CUH
PPH
GJ
HKP7
OH
SK
HKP1
HKP5
HKP6
STHI
HKP4
KW
KT
HKP3

## Slide 8
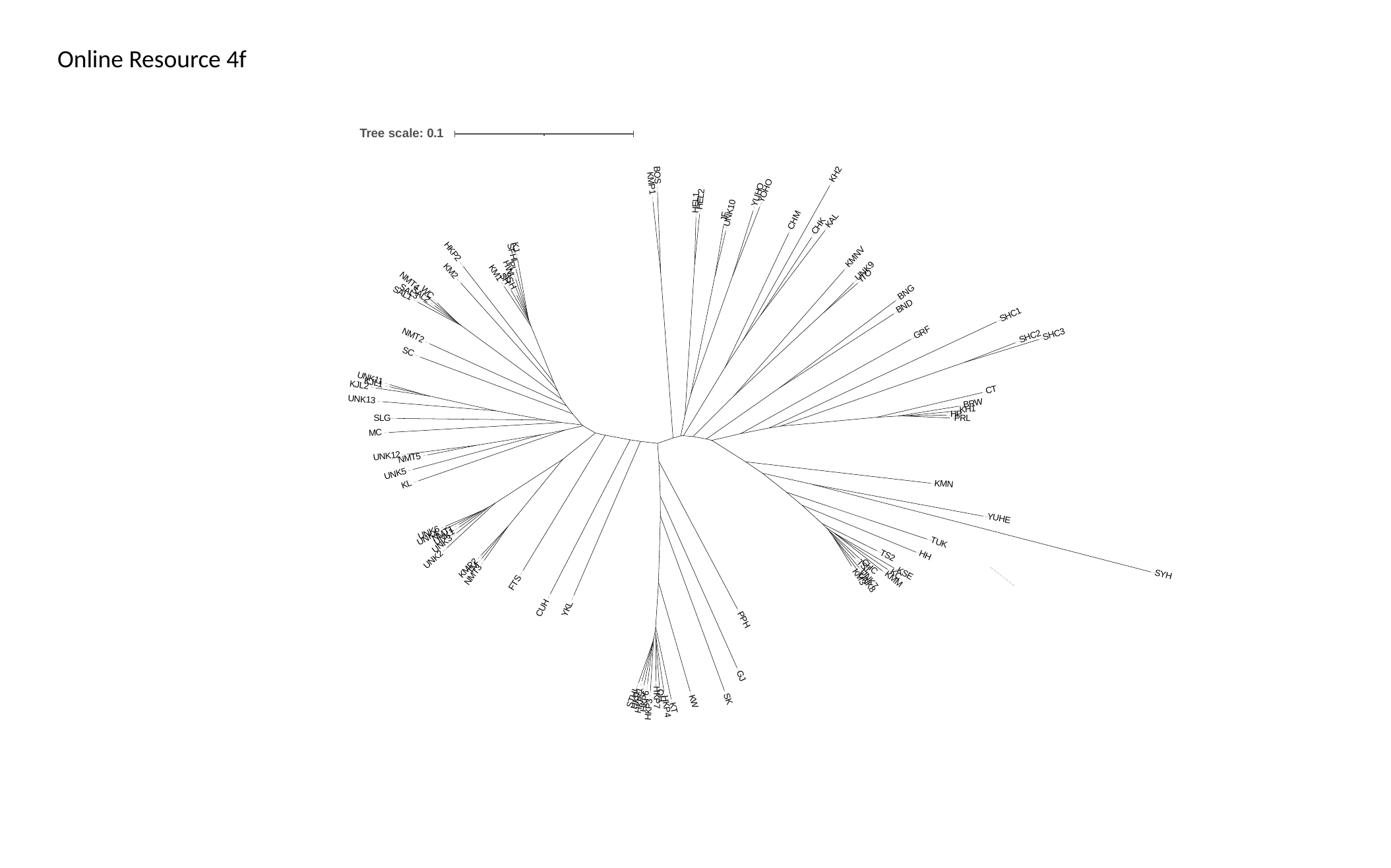

Online Resource 4f
Tree scale: 0.1
KH2
BOS
KMP1
YOHO
YUHO
HEL2
HEL1
UNK10
JF
CHM
KAL
CHK
KJ
HKP2
SFHI
KMNV
PL
UNK9
HWL
KM2
KM1
ITO
SH
NMT4
SSH
BNG
SAL3
WC
SAL1
SAL2
BND
SHC1
GRF
SHC3
SHC2
NMT2
SC
UNK11
KJL1
KJL2
CT
UNK13
BRW
KH1
HP
SLG
PRL
MC
UNK12
NMT5
UNK5
KL
KMN
YUHE
UNK6
NMT1
UNK1
UNK4
TUK
UNK3
HH
TS2
UNK2
TM
KMP2
TS1
CHC
NMT3
KSE
SYH
KC
KM3
UNK7
KMM
FTS
UNK8
CUH
YKL
PPH
GJ
HKP7
HKP1
STHI
OH
HKP6
SK
HKP5
KW
HKP4
HKP3
KT

## Slide 9
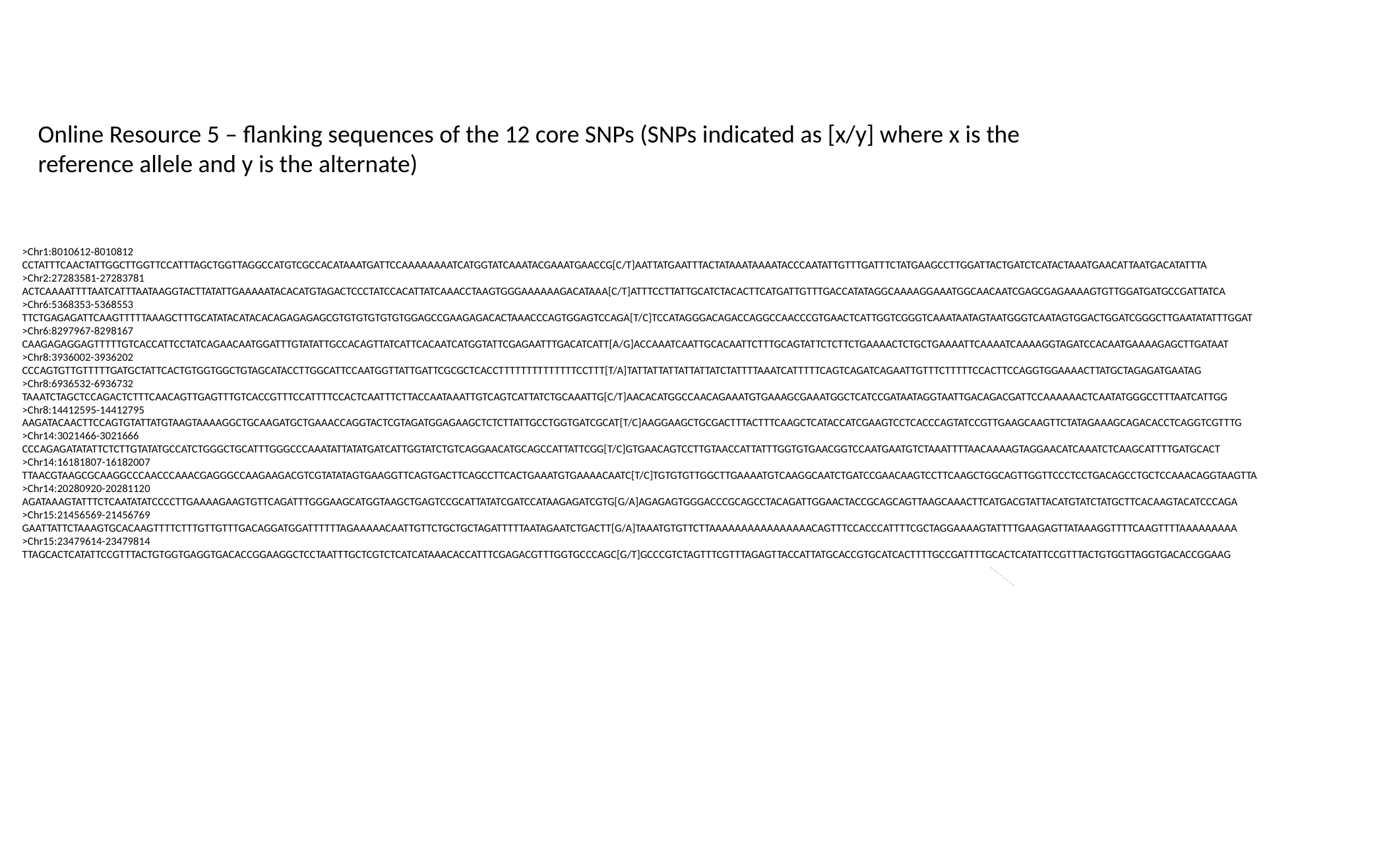

Online Resource 5 – flanking sequences of the 12 core SNPs (SNPs indicated as [x/y] where x is the reference allele and y is the alternate)
>Chr1:8010612-8010812
CCTATTTCAACTATTGGCTTGGTTCCATTTAGCTGGTTAGGCCATGTCGCCACATAAATGATTCCAAAAAAAATCATGGTATCAAATACGAAATGAACCG[C/T]AATTATGAATTTACTATAAATAAAATACCCAATATTGTTTGATTTCTATGAAGCCTTGGATTACTGATCTCATACTAAATGAACATTAATGACATATTTA
>Chr2:27283581-27283781
ACTCAAAATTTTAATCATTTAATAAGGTACTTATATTGAAAAATACACATGTAGACTCCCTATCCACATTATCAAACCTAAGTGGGAAAAAAGACATAAA[C/T]ATTTCCTTATTGCATCTACACTTCATGATTGTTTGACCATATAGGCAAAAGGAAATGGCAACAATCGAGCGAGAAAAGTGTTGGATGATGCCGATTATCA
>Chr6:5368353-5368553
TTCTGAGAGATTCAAGTTTTTAAAGCTTTGCATATACATACACAGAGAGAGCGTGTGTGTGTGTGGAGCCGAAGAGACACTAAACCCAGTGGAGTCCAGA[T/C]TCCATAGGGACAGACCAGGCCAACCCGTGAACTCATTGGTCGGGTCAAATAATAGTAATGGGTCAATAGTGGACTGGATCGGGCTTGAATATATTTGGAT
>Chr6:8297967-8298167
CAAGAGAGGAGTTTTTGTCACCATTCCTATCAGAACAATGGATTTGTATATTGCCACAGTTATCATTCACAATCATGGTATTCGAGAATTTGACATCATT[A/G]ACCAAATCAATTGCACAATTCTTTGCAGTATTCTCTTCTGAAAACTCTGCTGAAAATTCAAAATCAAAAGGTAGATCCACAATGAAAAGAGCTTGATAAT
>Chr8:3936002-3936202
CCCAGTGTTGTTTTTGATGCTATTCACTGTGGTGGCTGTAGCATACCTTGGCATTCCAATGGTTATTGATTCGCGCTCACCTTTTTTTTTTTTTTCCTTT[T/A]TATTATTATTATTATTATCTATTTTAAATCATTTTTCAGTCAGATCAGAATTGTTTCTTTTTCCACTTCCAGGTGGAAAACTTATGCTAGAGATGAATAG
>Chr8:6936532-6936732
TAAATCTAGCTCCAGACTCTTTCAACAGTTGAGTTTGTCACCGTTTCCATTTTCCACTCAATTTCTTACCAATAAATTGTCAGTCATTATCTGCAAATTG[C/T]AACACATGGCCAACAGAAATGTGAAAGCGAAATGGCTCATCCGATAATAGGTAATTGACAGACGATTCCAAAAAACTCAATATGGGCCTTTAATCATTGG
>Chr8:14412595-14412795
AAGATACAACTTCCAGTGTATTATGTAAGTAAAAGGCTGCAAGATGCTGAAACCAGGTACTCGTAGATGGAGAAGCTCTCTTATTGCCTGGTGATCGCAT[T/C]AAGGAAGCTGCGACTTTACTTTCAAGCTCATACCATCGAAGTCCTCACCCAGTATCCGTTGAAGCAAGTTCTATAGAAAGCAGACACCTCAGGTCGTTTG
>Chr14:3021466-3021666
CCCAGAGATATATTCTCTTGTATATGCCATCTGGGCTGCATTTGGGCCCAAATATTATATGATCATTGGTATCTGTCAGGAACATGCAGCCATTATTCGG[T/C]GTGAACAGTCCTTGTAACCATTATTTGGTGTGAACGGTCCAATGAATGTCTAAATTTTAACAAAAGTAGGAACATCAAATCTCAAGCATTTTGATGCACT
>Chr14:16181807-16182007
TTAACGTAAGCGCAAGGCCCAACCCAAACGAGGGCCAAGAAGACGTCGTATATAGTGAAGGTTCAGTGACTTCAGCCTTCACTGAAATGTGAAAACAATC[T/C]TGTGTGTTGGCTTGAAAATGTCAAGGCAATCTGATCCGAACAAGTCCTTCAAGCTGGCAGTTGGTTCCCTCCTGACAGCCTGCTCCAAACAGGTAAGTTA
>Chr14:20280920-20281120
AGATAAAGTATTTCTCAATATATCCCCTTGAAAAGAAGTGTTCAGATTTGGGAAGCATGGTAAGCTGAGTCCGCATTATATCGATCCATAAGAGATCGTG[G/A]AGAGAGTGGGACCCGCAGCCTACAGATTGGAACTACCGCAGCAGTTAAGCAAACTTCATGACGTATTACATGTATCTATGCTTCACAAGTACATCCCAGA
>Chr15:21456569-21456769
GAATTATTCTAAAGTGCACAAGTTTTCTTTGTTGTTTGACAGGATGGATTTTTTAGAAAAACAATTGTTCTGCTGCTAGATTTTTAATAGAATCTGACTT[G/A]TAAATGTGTTCTTAAAAAAAAAAAAAAAACAGTTTCCACCCATTTTCGCTAGGAAAAGTATTTTGAAGAGTTATAAAGGTTTTCAAGTTTTAAAAAAAAA
>Chr15:23479614-23479814
TTAGCACTCATATTCCGTTTACTGTGGTGAGGTGACACCGGAAGGCTCCTAATTTGCTCGTCTCATCATAAACACCATTTCGAGACGTTTGGTGCCCAGC[G/T]GCCCGTCTAGTTTCGTTTAGAGTTACCATTATGCACCGTGCATCACTTTTGCCGATTTTGCACTCATATTCCGTTTACTGTGGTTAGGTGACACCGGAAG
